# Embryonic organizer specification in the mud snail *Ilyanassa obsoleta* depends on intercellular signaling

**DOI:** 10.1242/dev.202027

**Published:** 2023-11-30

**Authors:** Jessica E. Wandelt, Ayaki Nakamoto, Morgan Q. Goulding, Lisa M. Nagy

**Affiliations:** ^1^ School of Biological Sciences, University of Texas at Austin, Austin, TX 78712, USA; ^2^ Faculty of Pharmaceutical Sciences, Aomori University, Koubata 2-3-1, Aomori 030-0943, Japan; ^3^ International Snail Station, Seattle, WA 98122, USA; ^4^ Department of Molecular and Cellular Biology, University of Arizona, Tucson, AZ 85721, USA

**Keywords:** Polar lobe, Axial patterning, Cell ablation, Intercellular signaling, Developmental evolution

## Abstract

In early embryos of the caenogastropod snail *Ilyanassa obsoleta*, cytoplasmic segregation of a polar lobe is required for establishment of the D quadrant founder cell, empowering its great-granddaughter macromere 3D to act as a single-celled organizer that induces ectodermal pattern along the secondary body axis of the embryo. We present evidence that polar lobe inheritance is not sufficient to specify 3D potential, but rather makes the D macromere lineage responsive to some intercellular signal(s) required for normal expression of 3D-specific phenotypes. Experimental removal of multiple micromeres resulted in loss of organizer-linked MAPK activation, complete and specific defects of organizer-dependent larval organs, and progressive cell cycle retardation, leading to equalization of the normally accelerated division schedule of 3D (relative to the third-order macromeres of the A, B and C quadrants). Ablation of the second-quartet micromere 2d greatly potentiated the effects of first micromere quartet ablation. Our findings link organizer activation in *I. obsoleta* to the putative ancestral spiralian mechanism in which a signal from micromeres leads to specification of 3D among four initially equivalent macromeres.

## Teaser

**Summary:** Polar lobe inheritance, long thought to autonomously specify the 3D organizer cell in *Ilyanassa*, surprisingly acts by sensitizing the D lineage to an organizer-inducing signal from multiple overlying micromeres.

## INTRODUCTION

Evolvability of body-patterning mechanisms at the earliest stages of embryogenesis is a longstanding problem of general interest. The metazoan superphylum known as Spiralia presents a unique opportunity for exploring this issue, with very distantly related taxa retaining conserved aspects of cleavage pattern intimately linked to early specification of body-region founder cells. Remarkably, the mechanism establishing one identified cell as a whole-body-patterning signaling center or ‘organizer’ seems to have undergone similar heterochronic shifts in numerous spiralian lineages (Freeman and Lundelius, 1992). In members of at least two phyla (annelids and mollusks), an evidently conserved early step of pattern formation involves an intercellular signal, mediated by ERK1/2, that selects the organizer cell (Lambert and Nagy, 2003; Seudre et al., 2022). Meanwhile, other lineages in both these phyla appear to have evolved a cell-autonomous mode of organizer specification, preempting intercellular ERK activation and accelerating establishment of the cell lineage of the organizer to the earliest possible stage of development. The present study reexamines one case where a transition from conditional to autonomous organizer specification is thought to have occurred.

In the pattern of spiral cleavage essentially conserved in multiple phyla, the first two mitotic divisions produce four body-quadrant founder cells (A, B, C and D), radially gathered around the axis marked by the site of polar body formation at the animal pole. These four cells then undergo three rounds of synchronous asymmetric division, successively budding three quartets of micromeres toward the animal pole, which are collectively fated to form the entire ectoderm. Between the three micromere quartets, which form staggered tiers encircling the animal pole, differentially inherited cytoplasmic factors confer distinct organ-forming potentials. This spatial pattern, together with the symmetric cluster of four remaining macromeres around the vegetal pole, creates an initial rudimentary whole-body organization along the primary (animal-vegetal) embryonic axis. Subsequently overlaid on this pattern is a gradient of intercellular signaling along an orthogonal secondary axis, initiated by one ‘organizer’ cell of the D lineage. This two-step body-patterning mechanism was revealed by experiments on distantly related gastropod mollusks (Arnolds et al., 1983; Clement, 1976; Lambert, 2010; van den Biggelaar, 1977): in every snail taxon examined, organizer activity begins in the 3D macromere (or its daughter 4d) (Clement, 1962; Henry et al., 2006; Labordus and van der Wal, 1986; Martindale, 1986), and is linked to activation of an ERK1/2-type mitogen-activated protein kinase (MAPK) exclusively in the 3D macromere (hereafter referred to simply as 3D) (Henry and Perry, 2008; Koop et al., 2007; Lambert and Nagy, 2001; 2003; Tan et al., 2022).

Spiralians that conditionally activate the organizer share an evidently primitive style of ‘equal spiral cleavage’, in which radial symmetry about the primary embryonic axis is maintained until after formation of the third micromere quartet. In these embryos, symmetric cell divisions in the first two rounds of mitosis produce macromeres that show no difference in potential upon experimental challenge. Within each of the three subsequently formed micromere quartets, all four cells likewise appear intrinsically equipotent. Following third quartet formation, equivalence of the four third-order macromeres is broken when one of them adopts the identity of 3D, activates ERK1/2 MAPK, and initiates organizer signaling that patterns the ectodermal micromere array. The crucial selection of just one macromere as 3D is mediated by stochastically directional juxtacrine signaling from any or all of the four first-quartet micromere derivatives (precursors of head ectoderm) that surround the animal pole. Ablation of one or two selected first-quartet cells imposes a strong spatial bias on 3D selection among the macromeres (Arnolds et al., 1983; van den Biggelaar and Guerrier, 1979); furthermore, 3D specification can be entirely blocked by killing the whole first quartet (Boring, 1989; Freeman and Lundelius, 1992; Henry et al., 2006; van den Biggelaar and Guerrier, 1979). Evidence for inductive action of first-quartet micromeres in 3D specification has also been obtained by experiments in an early-branching mollusk (van den Biggelaar, 1996) and in three other spiralian phyla [platyhelminths (Boyer, 1989), nemerteans (Henry, 2002) and annelids (Gonzales et al., 2007; Seudre et al., 2022)].

In those mollusks and annelids believed to activate the organizer cell-autonomously, various alternative modes of ‘unequal spiral cleavage’ occur. Generally, asymmetric divisions partition distinct cytoplasmic materials among the four body-quadrant founder cells; in nearly all of these cases, extra cytoplasm is inherited by the D quadrant founder cell (the great-grandmother of 3D) (Freeman and Lundelius, 1992). Such a cell-autonomous mechanism of specifying the organizer appears to have evolved near the base of the very large snail clade Caenogastropoda. The first experimental evidence of precocious D quadrant specification was provided by seminal experiments on embryos of the mud snail *Ilyanassa obsoleta* (Crampton and Wilson, 1896). During each of the first two cleavages in *Ilyanassa* embryos, a transiently extruded ‘polar lobe’ (PL) shunts material from the vegetal region of the egg to one daughter cell; PL inheritance endows the D lineage with its unique early cell division pattern as well as the unique ability to induce ectodermal pattern (Clement, 1952). PL inheritance in *Ilyanassa* has since been presumed sufficient to determine all aspects of D lineage fate; in contrast with ‘equal-cleaving’ spiralians, no extracellular signal appeared to act in 3D specification. Consistent with this view, ablation of all four first-quartet micromeres resulted in development of headless but otherwise perfect larvae (Sweet, 1998), indicating undisturbed organizer function and implying cell-autonomous specification of 3D. In another caenogastropod, *Bithynia*, first-quartet ablation had the same result (van Dam and Verdonk, 1982). However, in yet another caenogastropod, *Crepidula*, 3D specification depends on the presence of first-quartet cells but not strictly on PL inheritance (Henry et al., 2006, 2017).

We set out to more rigorously test the possibility that 3D specification in *I. obsoleta* requires signaling from micromeres. Using four independent assays of 3D identity or organizer function, we assessed the effect of ablating single and multiple micromeres in isolated D quadrants as well as in otherwise intact embryos. Surprisingly, all assays indicated that 3D specification depends on intercellular signaling as well as PL inheritance. We also unexpectedly found that the 3D-specifying signal from multiple first-quartet cells is joined by a signal from at least one second-quartet cell (namely 2d), and the absence of one signal source could be compensated by others. Thus, it appears that conditional 3D specification in *Ilyanassa* was previously obscured by the additive action of multiple signaling cells.

## RESULTS AND DISCUSSION

### PL inheritance is required for MAPK activation in a third-order macromere

**Fig. 1.**
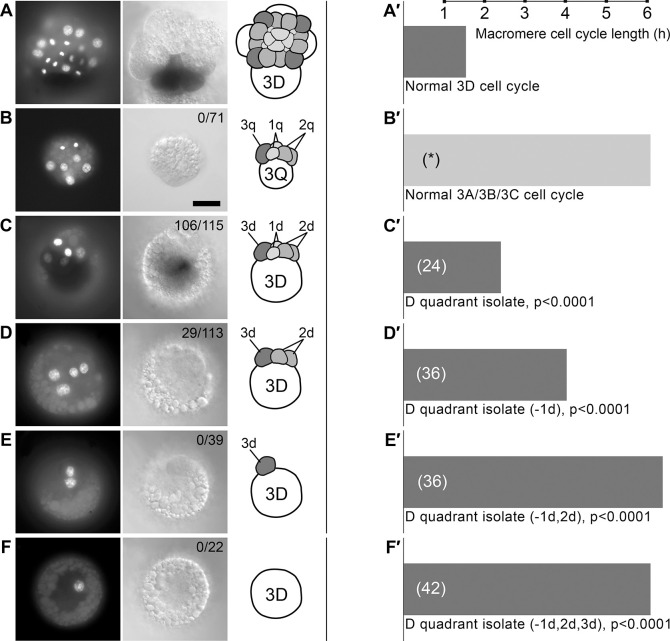
**Micromere ablation effects on 3D MAPK activation and cell cycle timing in quadrant isolates.** (A-F) Sibling whole and partial embryos assayed for activated MAPK, fixed 80 min after formation of the third micromere quartet. Left: DAPI-stained representative embryos. Middle: differential interference contrast (DIC) images of the same embryos showing activated MAPK detected with HRP. Right: cartoons showing the cells present. The numbers of embryos expressing active MAPK relative to the total number of embryos analyzed are indicated. (A) Activated MAPK in an intact control embryo. (B) Absence of activated MAPK in unidentified non-D quadrant isolate (A,B or C quadrant) (*P*<0.0001). (C) MAPK activation in D quadrant isolate (*P*<0.20). (D) Absence of activated MAPK in D quadrant isolate lacking 1d (*P*<0.0001). (E) Absence of activated MAPK in D quadrant isolate lacking 1d and 2d (*P*<0.0001). (F) Absence of activated MAPK in D quadrant isolate lacking 1d, 2d and 3d (*P*<0.0001). (A′-F′) Bars representing median cell cycle length (time to fourth micromere formation) in classes of control and operated embryos shown in A-F. The numbers of embryos in each sample are indicated in parentheses. The asterisk (*) in B signifies that 3Q division timing in non-PL-inheriting quadrant isolates was not measured in this study; the value shown represents cell cycle duration in PL-deleted embryos (Clement, 1952). *P*-values for cell cycle length comparisons are results of a two-tailed Mann–Whitney *U*-test. Scale bar in B: 50 μm.

In early embryos of *I. obsoleta*, reactivity to an antibody against the kinase-active form of MAPK (ERK1/2) first appears in the 3D macromere shortly after its birth and persists until the cell divides about 90 min later; no other cells express this immunoreactivity at these or earlier stages. Pharmacological interference with the earliest period of MAPK activation blocks ectodermal organogenesis that is normally induced by the 3D organizer (Lambert and Nagy, 2001). We therefore focused on the expression of active MAPK in 3D as a proxy for activation of organizer function. First, we tested the assumption that 3D MAPK activation depends on PL inheritance by the D cell at the second cleavage. When quadrant founder cells (A, B, C or D) were cultured after isolation at the four-cell stage and immunostained for active MAPK about 80 min after formation of their third micromeres, only the 3D cell showed a positive result, as expected (Fig. 1A-C). We also observed absence of MAPK activation in any cells of unidentified quadrants isolated after PL removal at first cleavage (*n*=71).

### MAPK activation in isolated D quadrants depends on the presence of micromeres

Aiming to identify possible extracellular influence on 3D organizer function, we examined MAPK activation in an idealized experimental setup with a minimum number of cells present. D quadrant founder cells isolated at the four-cell stage proceed through cleavage and subsequent development with only minor deviation from *in situ* behavior (Clement, 1956; Crampton and Wilson, 1896; Goulding, 2003). To assess the autonomy of 3D organizer function, we repeated the isolation of D quadrant founder cells, then additionally removed one or more micromeres (1d/2d/3d), subsequently assaying for activation of MAPK in 3D (Fig. 1D-F). In the great majority of whole D quadrant isolates (106/115), MAPK was activated as normal in the 3D macromere. Following ablation of the 1d micromere, however, only 29/113 D quadrant isolates showed MAPK activation in 3D (Fig. 1D). Successive removal of both first- and second-quartet micromeres (*n*=39) or all three micromeres (*n*=22) abolished 3D MAPK activation in every case (Fig. 1E,F).

### Normal precocity of 3D mitosis depends on presence of micromeres

Another conserved aspect of 3D behavior is a cell division pattern distinct from that of its cousins in the A, B, and C quadrants. Following MAPK activation, 3D uniquely divides to produce the mesentoblast 4d (founder of most of the mesoderm, as well as the hindgut). In *I. obsoleta,* this division occurs 3-4 h before the synchronous division of the 3A, 3B and 3C (collectively, 3ABC) macromeres (Clement, 1952; Goulding, 2009). PL removal eliminates this difference, causing all 3Q cells (collectively referring to 3A, 3B, 3C and 3D) to divide with the long delay of 3ABC macromeres (and yielding all non-mesentoblast daughters) (Clement, 1952). While examining D quadrant isolates for MAPK activation, we noticed that 3D division was retarded in every class of partial embryo. We therefore examined division timing closely in another set of experiments. In D quadrant isolates cultured intact with all D quadrant micromeres (*n*=24), 3D divided approximately 50 min later than 3D in whole embryos from the same brood (Fig. 1A′-C′). In D quadrant isolates lacking the 1d cell (*n*=36), the abnormal 3D cell cycle delay was prolonged to almost 150 min (Fig. 1D′). Following ablation of both 1d and 2d cells (*n*=36), 3D division was delayed by almost 300 min (Fig. 1E′); in this situation, 3D division coincided with the timing of 3ABC division in whole embryos. The same long delay of 3D division resulted after ablation of all three micromeres (*n*=42) (Fig. 1F′).

**Fig. 2.**
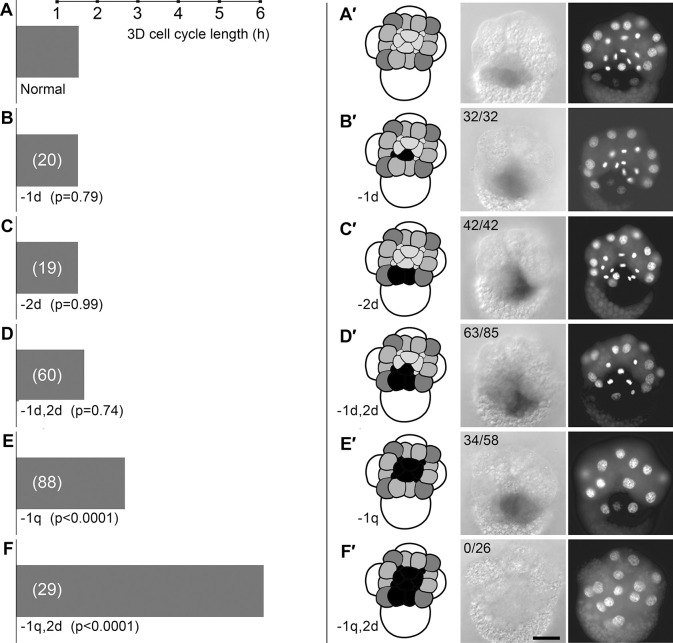
**3D cell cycle timing and MAPK activation depend on multiple micromeres.** (A-F) Bars representing median 3D cell cycle length in classes of control and operated embryos shown in A′-F′. The numbers of embryos in each sample are indicated in parentheses. *P*-values for cell cycle length comparisons are results of a two-tailed Mann–Whitney *U*-test. (A′-F′) Co-cultured sibling whole and partial embryos assayed for activated MAPK, fixed 80 min after formation of the third micromere quartet. Left: cartoons showing cells present, with ablated micromeres colored black. Middle: DIC images of the same embryos showing activated MAPK detected with HRP. Right: DAPI-stained representative embryos. The numbers of embryos expressing active MAPK relative to the total number of embryos analyzed are indicated. Scale bar: 50 μm.

We next examined 3D division timing after removal of selected micromeres from whole embryos (Fig. 2A-F). Ablation of either 1d (*n*=20) or 2d (*n*=19) or both 1d and 2d (*n*=60) had little effect on 3D division timing; in all cases, 3D divided within 20 min of the division timing of controls. By contrast, ablation of the entire first quartet (1a, 1b, 1c and 1d, collectively abbreviated ‘1q’) resulted in prolongation of the 3D cell cycle by approximately 70 min (*n*=88). Strikingly, ablation of 1q and then 2d resulted in a 3D division delay of nearly 5 h (*n*=29), coinciding with division of 3ABC macromeres in the same embryos as well as in unoperated controls.

### Synergistic effects of micromere ablations on MAPK activation in 3D

Given the different effects observed after ablation of 1d and 2d in whole embryos compared with the same double ablation in D quadrant isolates, we extended the assay of 3D MAPK activation by removing single or multiple micromeres from whole embryos. Ablation of 1d (*n*=32) or 2d (*n*=42) had no apparent effect on 3D MAPK activation (Fig. 2A′-C′). Successive ablation of both 1d and 2d resulted in detectable 3D MAPK activation in only 74% of embryos (*n*=85, Fig. 2D′). Following ablation of the entire first quartet (1q), 3D MAPK activation was detectable in 59% of embryos (*n*=58, Fig. 2E′). Again, ablation of both 1q and 2d yielded a much stronger effect, resulting in absence of 3D MAPK activation in all embryos examined (*n*=26, Fig. 2F′).

### 3D-induced ectodermal MAPK activation depends extrinsically on 1q and 2d

**Fig. 3.**
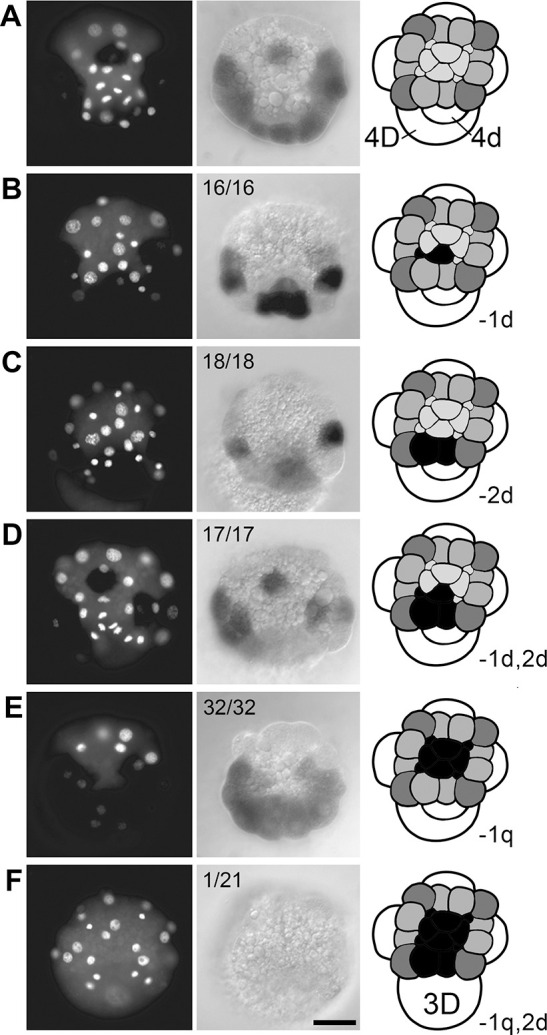
**Ectodermal MAPK activation after early micromere ablations.** Sibling whole and partial embryos assayed for activated MAPK, fixed 210 min after formation of the third micromere quartet. (A) Control embryo. (B) Embryo after 1d ablation. *n*=16. (C) Embryo after 2d ablation. *n*=18. (D) Embryo after ablation of 1d and 2d. *n*=17. (E) Embryo after ablation of entire first quartet (1q). *n*=32. (F) Embryo after ablation of 1q and 2d. The cartoons depict ablated cells in black. The numbers of embryos expressing active MAPK relative to the total number of embryos analyzed are indicated. Note the presence of the 4d cell in A-E. MAPK activity was observed in remaining derivatives of second- and third-quartet cells in the A,C and D quadrants, with no activation observed in derivatives of 2b or in the third-quartet cells 3a and 3b. Cell positions were shifted after micromere removal, obscuring more precise definition of the micromere MAPK activation pattern (B-E). Scale bar: 50 μm.

After MAPK activation has begun in 3D, the same phosphoepitope appears progressively in ectodermal precursors of all three micromere quartets, beginning with the midline 2d daughters and spreading bilaterally throughout the subset of cells that have 3D-dependent fates; the fates of these cells are also sensitive to an inhibitor pulse during the time interval of their own MAPK activation. This ectodermal MAPK activation depends strictly on PL inheritance by the D lineage, and specifically on the presence of 3D (Lambert and Nagy, 2001). We therefore asked whether ectodermal MAPK activation is sensitive to early removal of the 1q and 2d micromeres. Following ablation of just the 1d (*n*=16) or 2d (*n*=18), all embryos showed activated MAPK in the remaining ‘dorsal’ and lateral micromeres (Fig. 3A-C). The same was seen in all embryos lacking both 1d and 2d (*n*=17) (Fig. 3D) or the entire first quartet (*n*=32) (‘1q’ in Fig. 3E). The latter finding is surprising, considering that nearly half of the 1q-ablated embryos did not activate 3D MAPK; this discrepancy suggests that the wave of organizer-dependent MAPK activation in micromeres may result from a 3D-derived signal that is independent of MAPK activation in 3D itself.

Successive removal of 1q and 2d had a much stronger effect on ectodermal MAPK activation than either ablation alone, with detectable micromere MAPK activation abolished in 20/21 embryos (Fig. 3F). The correlation with the apparently synergistic influences of 1q and 2d on both MAPK activation and cell division timing in 3D suggests that the 1q and 2d ablation disrupts micromere MAPK activation through a primary defect in the 3D cell, although an additional direct influence of 1q and 2d on micromere MAPK activation cannot be ruled out.

### Distinct effects of multiple micromere ablations on trunk ectoderm organogenesis

Extending the assessment of organizer function, we followed ablation of first- and second-quartet cells by culturing embryos to near-hatching larva stage, assaying development of organizer-dependent trunk ectoderm (shell and foot). The foot of hatching-stage veligers, formed by parts of the 2a, 2c and 2d clones as well as most descendants of 3c and 3d (Render, 1997), is distinguished by a bilateral pair of statocysts, an operculum covering most of the dorsal surface, and a tapered posterior end with terminal setae; none of these structures ever develops in PL-ablated embryos (Atkinson, 1971; Clement, 1952, 1962). The shell of the veliger, formed by parts of all four 2q clones (Render, 1997), winds roughly one full turn by the hatching stage. Following organizer ablation, a shell never develops, but most embryos form several masses of ‘internal shell’ enclosed in misplaced shell gland tissue (Atkinson, 1971; Clement, 1952, 1962; Moshel et al., 1998). Also worth noting is that in the absence of a functional 3D organizer, the post-gastrulation morphogenesis of the embryo is radialized. Normally, the ectodermal territory centered around the meridian defined by the 2d clone undergoes elongation parallel to that meridian, and this stretched-out tissue gives rise to both shell and foot (see cartoon in figure 1 of Goulding, 2009). No such trunk elongation occurs in the absence of an organizer, and no secondary body axis can be distinguished (Atkinson, 1971; Clement, 1952).

**Fig. 4.**
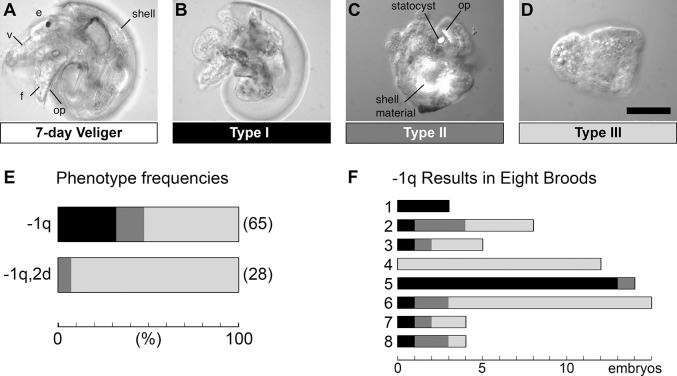
**Ablation of multiple micromeres disrupts organizer-induced organogenesis, with effects varying between broods.** (A-D) Representative images of experimental embryos scored for larval morphogenesis and differentiation at 7 days of development. (A) Unoperated control; near-hatching-stage veliger. Left side view, anterior to the left, showing one of the eyes (e), velum (v), foot (f) with operculum (op), and shell. (B) Type I embryo. Left side view, anterior to the left. The trunk structures appeared normal and head structures were absent. (C) Type II embryo with no evident dorsoventral axis. Several tissue structures (internal shell mass, partial operculum and statocyst) were revealed by birefringence under polarized light. (D) Type III embryo with no dorsoventral axis and no evident organs. (E) Phenotype frequencies after ablation of the entire first quartet (−1q) or sequential ablation of 1q and 2d (−1q,2d). Phenotypes are differentially shaded: Type I is black, Type II is dark gray and Type III is pale gray. Total numbers of embryos are indicated. (F) Numbers of 1q-ablated embryos showing each phenotype in eight broods. Phenotypes are color-coded as in E. Scale bar: 100 μm.

Fig. 4A-D shows hatching-stage embryos of unoperated controls and three distinct phenotypes resulting from ablation of multiple micromeres. After 1q ablation, a third of all operated embryos (21/65) developed a flawless trunk and a complete absence of head structures (i.e. normally 1q-derived tissue), as previously reported (Sweet, 1998). We refer to this ‘headless’ phenotype, which indicates full functioning of the 3D organizer, as Type I. In another 15% of 1q-ablated embryos (Type II), shell and foot structures developed incompletely and with deranged spatial organization. Strikingly, most 1q-ablated embryos (52%) formed neither shell/foot structures nor a recognizable body axis; this phenotype (Type III) indicates complete failure of organizer signal reception. Because of their extreme malformations, we refer to these embryos as Type III ‘monsters’. Type III embryos did consistently display localized morphological differentiation of the ectodermal epithelium, indicating that they did not suffer from a complete cell differentiation blockade. Once again, successive ablation of 1q and 2d resulted in much more penetrant disruption of organizer function, with Type III monsters developing in almost every case (26/28), and the remainder developing as Type II (Fig. 4E). All three phenotypes were observed following successive ablation of 1q and either 2a, 2b or 2c (*n*=26 for each), with Type II being the most common in each case; none of these results differed significantly from results of 1q ablation (Tables S1 and S2).

Considering patterned ectodermal differentiation as the definitive readout of organizer function, its success is interestingly correlated across experimental conditions with MAPK activation in 3D, but not with MAPK activation in ectodermal precursors. This relationship supports the notion of a key role for MAPK activation in 3D, which evidently predates the divergence of mollusks and annelids (Lambert and Nagy, 2003; Seudre et al., 2022). The subsequent MAPK activation in micromeres, which has so far been observed only in *Ilyanassa*, appears to be necessary but not sufficient for determining organizer-dependent trunk ectoderm fates, implying the action of a parallel signal from the 3D macromere that is not mediated by MAPK in micromeres.

It should be noted that Type III monsters displayed an ectodermal defect beyond the worst-case scenario resulting from PL removal. ‘Lobeless’ embryos usually form disorganized masses known as ‘internal shell’ (Atkinson, 1971; Clement, 1952), with mineral composition similar to that of normal larval shell (McCain, 1992); these masses also develop following D macromere ablation (Clement, 1962) and in isolated non-D quadrants (Clement, 1956). Internal shell was found in most of our Type II embryos, but in none of the Type III embryos. This evident dependence of (abnormal) biomineralization on the early presence of 1q and 2d is surprising and may reflect serendipitous disruption of another unknown intercellular signal. Biomineralization has been shown to depend on the presence of any macromere (Cather, 1967); in light of this fact, our result raises the intriguing possibility that micromeres induce a biomineralization-promoting power of non-D macromeres at the same time they induce organizer function in 3D.

Returning to the question of 3D induction, the additional demonstration by Cather (1967) that a grafted PL can serve as the organizer to an isolated ectoblast implies, in the light of our findings, that the responsiveness of the 3D cell to the organizer-inducing signal from micromeres is a property inherent to the vegetal cytoplasm, and further implies that this property is independent of zygotic gene transcription.

### Effect of 1q ablation on trunk ectoderm development varies between broods

One of our results disagrees with data shown in a previous study of *I. obsoleta*, in which 1q ablation yielded only headless larvae with well-formed trunks (Sweet, 1998). In our experiments in which larval organogenesis was assayed, 1q ablations were done on embryos from eight broods collected on different days, very probably from different mothers; importantly, our results showed large variation between broods (Fig. 4F). For example, 1q ablation abolished organizer-dependent trunk ectoderm development in all 12 operated embryos from one brood, whereas in another brood, nearly all 1q-ablated embryos (13/14) developed as headless but otherwise well-formed veligers. Such interbrood variation is likely to blame for Sweet's failure to observe an organizer defect among the embryos challenged with 1q ablation in their study (*n*=16). Possible (and not mutually exclusive) causes of interbrood variation include genetic difference among wild snails, phenotypic plasticity in response to maternal or embryonic stress and, simply, stochastic fluctuation in micromere signaling or 3D receptivity. It cannot be ruled out that very small differences in the timing of micromere ablations in the present study contributed to the variation observed.

### 3D MAPK activation as an ultrasensitive response to graded input from multiple cells

Our data support a model in which 3D specification depends on signaling from multiple micromeres simply acting in an additive way, and normally summing to surpass an activation threshold; 1q ablation may lower the signal level approximately to the threshold. We observed clear presence or absence of activated MAPK in 3D, with no evident intermediate states. This is consistent with an ultrasensitive (all-or-none) MAPK response to a threshold level of signal (Ferrell, 1996).

We were unable to judge the relative contributions of identified cells to 3D induction. In D quadrant isolates, the combination of 1d and 2d appeared necessary and sufficient to activate 3D MAPK. However, loss of these two cells in otherwise intact embryos appeared to be compensated by other cells. How many cells normally signal to 3D, and which ones? The occasional success of 3D specification in 1d-ablated D quadrant isolates weighs against a requirement for a minimum total number of micromeres greater than the three present in one quadrant. This fact, along with the variable results of 1q ablation in whole embryos, also rules out a strict requirement for first-quartet cells per se; whatever the contribution of the first quartet to organizer induction might be, its absence can (sometimes) be compensated by the 2d cell, possibly with help from other cells.

### Evidence for separable responses to micromere signaling

As well as suggesting that 3D induction involves signaling from multiple cellular sources, our results point to separable downstream effects. In contrast to the all-or-none 3D MAPK response, the lengthening of the 3D cell cycle observed in both D quadrant isolates and micromere ablations in whole embryos increased progressively with the removal of more cells. Unlike MAPK activation, cell cycle delay was observed in all D quadrant isolates, indicating that mitotic timing demands additional signaling beyond the threshold needed for MAPK activation. The distinct responses of MAPK activation and cell cycle control could be a clue to independent signal transduction pathways, potentially leading to distinct aspects of 3D development (i.e. organizer function and mesentoblast formation).

### An unexpected role for 2d

The present study is the first to document a 3D-inducing role for any non-first-quartet cell in a spiralian. In *Ilyanassa,* juxtacrine signaling from 2d is certainly favored by its position (more accurately, that of its daughters 2d^1^ and 2d^2^) with a large area of direct contact to 3D. However, the daughters of 2a and 2c are also in direct (albeit slimmer) contact with 3D (Sweet, 1996). Our results leave open the question of whether the 2d micromere has an intrinsically special property compared with other second-quartet micromeres. In other spiralian taxa with early D quadrant establishment, 2d is indeed intrinsically special (obviously so, by inheriting much more cytoplasm than other second-quartet cells), but such has not been shown in any gastropod. The contribution of 2d and/or other cells to 3D induction in *I. obsoleta* may not be unique: Boring (1989) reported a large minority of 1q-ablated embryos developing shell and/or foot structures in a cephalaspidean opisthobranch with equal cleavage. Future experiments should be done to identify all the sources of 3D-inducing signal in *Ilyanassa* and other spiralian embryos.

### *Ilyanassa* is more primitive than supposed

PL segregation into the D quadrant has apparently evolved in three molluscan lineages: in bivalves, in scaphopods and near the base of the hyperdiverse snail clade Caenogastropoda (Cunha and Giribet, 2019; Freeman and Lundelius, 1992). Experiments in two other caenogastropod taxa indicate surprising variation in PL function. In *Bithynia*, PL removal abolished organizer-dependent ectodermal fates (Cather and Verdonk, 1974), whereas 1q ablation yielded complete trunks lacking heads (van Dam and Verdonk, 1982). In this case, PL inheritance indeed appears sufficient for 3D specification (although the present study casts doubt on this conclusion). In *Crepidula*, by contrast, 1q ablation abolished 3D MAPK activation and organizer activity, whereas PL removal did not block organizer specification but rather randomized it to one 3Q cell or another (or sometimes more than one) (Henry et al., 2006, 2017); furthermore, ablation of the presumptive D macromere descended from the PL-inheriting quadrant founder cell in *Crepidula* resulted in functional replacement by another macromere (Henry et al., 2006). It seems that in different caenogastropod lineages, an ancestral mechanism conditionally specifying 3D has been supplemented or supplanted by segregated factors in the vegetal cytoplasm that bias selection to varying degrees. The cell-autonomous choice may have taken over completely in *Bithynia*; in *Crepidula*, PL material merely offers a strong but tentative suggestion. In *I. obsoleta*, the bias is absolute, but intercellular signaling is still required. Together with the surprises from *Crepidula*, our findings give reason to question the assumption that intrinsic inequality indicates differential fate determination.

Indeed, we would argue that it is time to dismiss the simplistic dichotomy of equal versus unequal cleaving spiralians and to experimentally test the autonomy of D quadrant specification in mollusks and annelids with PLs and/or unequal-sized quadrant founder cells. The straightforward approach of cell ablation within respective phylogenetic contexts may reveal stepwise transitions from conditional to autonomous specification in multiple clades; it will be interesting to discover commonalities and disparities of molecular mechanism in the parallel trials of this long-running natural experiment. Notable questions of current interest include asking how 3D MAPK activation has come to be lost (and how it has been replaced) in multiple annelid lineages (Seudre et al., 2022), how organizer function has shifted from 3D to another D quadrant cell (Henry et al., 2017; Amiel et al., 2013), whether FGF signaling from micromeres is the conserved proximate cause of 3D MAPK activation (Seudre et al., 2022) and whether cell-autonomous MAPK activation could alternatively be at play (Chessel et al., 2023), as well as how ancient axis-patterning SMAD function may or may not be conserved in spiralian organizer signaling (Lambert et al., 2016; Lanza and Seaver, 2018, 2020; Lyons et al., 2020; Tan et al., 2022; Webster et al., 2021). A key to all these questions would be provided by discovering, in diverse taxa displaying 3D MAPK activation, the immediate molecular causes and consequences of this primitive and developmentally pivotal event.

## MATERIALS AND METHODS

### Snails and embryo culture

*Ilyanassa obsoleta* adults obtained from the Marine Resources Center (Marine Biological Laboratories, Woods Hole, MA, USA) were kept in 76 l tanks (ASW, Instant Ocean) with 3 cm of aquarium rock substrate, sponge filtration and bubbler aeration at room temperature (RT, 23±1**°**C) and fed frozen clam meat every other day. Embryos were collected from newly deposited egg capsules as described previously (Collier, 1981), and cultured at 23±1**°**C in groups of three to 15 in 35 mm plastic Petri dishes with 2-3 ml of 0.2 μm-filtered artificial sea water (FASW, Instant Ocean). For longer incubation, penicillin (100 U/ml) and streptomycin (200 μg/ml) were added, and embryos were moved to fresh FASW in a new dish every 2 days (Gharbiah et al., 2009).

### Microsurgery

For C or D quadrant isolations, the AB cell (mother of the A and B quadrant founder cells) was lysed by needle puncture during the early two-cell stage, and its cellular remnants removed; this was followed by lysing and removing either the D cell or the C cell soon after completion of second cleavage. To isolate the A or B quadrants, we lysed the CD cell (mother of the C and D quadrant founder cells) in the same manner at the two-cell stage, and then lysed one of the two indistinguishable remaining cells (A or B) shortly after their formation at second cleavage. For PL(−) quadrant isolations, PLs were removed at the trefoil stage (first cleavage) by gentle agitation in low-Ca^2+^/Mg^2+^ FASW (Collier, 1981; Lambert and Nagy, 2001). Under these conditions, the PL spontaneously separates and falls away upon full constriction of the furrow. Following PL removal, one cell at the two-cell stage was lysed by needle puncture and removed as described above, followed by another cell lysis and removal after the second cleavage. Micromere ablations were carried out during a narrow time window following the formation of the micromere quartet (10-25 min after apparently full constriction of cytokinetic furrows, equivalent to the interval spanning 20-25% of the cell cycle). Micromeres were removed in rapid succession during this time window; after this time interval, compaction makes micromeres very hard to lyse by freehand needle puncture. Lysed cells invariably detach from the embryo within minutes, and are easily removed by gentle pipetting.

### Cell cycle analysis

The median timing of 3D division (i.e. fourth micromere formation) was determined by the following method: pools of micromere-ablated and control embryos were kept in separate small Petri dishes and monitored every 15-20 min. The time when the first cells had divided in each dish was recorded, followed by the time when cell division had begun in roughly half the population. The ranges of cell division timing did not greatly exceed the normal range (roughly 30 min). Statistical significance of the differences in cell cycle length was calculated using the Mann–Whitney *U*-test (https://www.socscistatistics.com/tests/mannwhitney/default2.aspx).

### Immunohistochemistry

MAPK activation was detected with a mouse monoclonal antibody (Sigma-Aldrich, M8159) recognizing a diphosphorylated 11-amino-acid peptide in the conserved activation loop of ERK1/2, as previously described (Lambert and Nagy, 2001, 2003). Briefly, embryos were fixed in 3.7% formaldehyde in 90% FASW for 30-35 min. Embryos were dehydrated and stored in methanol at −20°C. Embryos were rehydrated by three 10-min washes in PBS containing 0.1% Tween 20 (PBTw), blocked for at least 1 h in PBTw containing 2% bovine serum albumin (PBTw+BSA), then incubated for 8-16 h at 4**°**C in primary antibody diluted in PBTw+BSA. After primary incubation, embryos were washed six times for 10 min in PBTw, then incubated with anti-mouse horseradish peroxidase (HRP)-linked secondary antibody (1:500; AB_10015289, 115-035-003, Jackson Immunoresearch) for at least 2 h (room temperature) with gentle agitation on a shaking platform, washed as above, and detected with Pierce HRP detection kit (Thermo Fisher Scientific) according to the manufacturer's instructions. After HRP detection, embryos were incubated in DAPI (Molecular Probes; 1 μg/ml) for 15 min (room temperature), then washed three times quickly in PBTw. PBTw was replaced with 80% glycerol:1× PBS for imaging. Differences in the frequency of MAPK activation between experimental groups were assessed using the χ^2^ test.

### Image acquisition and analysis

In immunohistochemical preparations, the cell nuclei were visualized by DAPI staining. Seven-day-old embryos (veligers) were relaxed in a 1:1 mixture of FASW and saturated aqueous chlorobutanol (Clement and Cather, 1957; Gharbiah et al., 2013), then fixed by the same method used for early embryos. Polarizing optics were used to visualize birefringent materials (shell, statocysts, operculum and internal shell). Other larval structures (esophagus, stomodeum, intestine, retractor muscle, eye and velum) were identified according to previous descriptions (Atkinson, 1971; Clement, 1952; Sweet, 1998). Fixed embryos were imaged with a Zeiss Axioskop using epifluorescence, differential interference contrast and polarizing optics. Cell divisions in living embryos were observed with a Zeiss dissecting stereomicroscope.

## Supplementary Material

10.1242/develop.202027_sup1 Supplementary information
