# Supplementary material for: Embryonic organizer specification in the mud snail Ilyanassa obsoleta depends on intercellular signaling
Source: Development. 2023 Nov 30;150(23):dev202027. doi: 10.1242/dev.202027 (PMC10730015; doi:10.1242/dev.202027)
Supplement: Supplementary information [file develop-150-202027-s1.pdf]

Table S1. Phenotypes of micromere ablations.

ablated micromeres	−1q	−1q−2a	−1q−2b	−1q−2c	−1q−2d	−1q (Sweet, 1998)
Number of capsules	8 capsules	3 capsules	4 capsules	4 capsules	4 capsules	?
Type I, D-V axis, coiled shell	21	7	5	2	0	16
Type II, no D-V axis, some tissues	10	10	13	13	2	0
Type III, no tissues, cell mass - monsters	34	9	8	11	26	0
Total	65	26	26	26	28	16

Table S2. Steel-Dwass test (calculated by R) on micromere ablations.

Ablation types compared	p-value	significance
−1q−2a : −1q−2b	9.99E-01	N.S
−1q−2a : −1q−2c	6.14E-01	N.S
−1q−2a : −1q−2d	4.33E-05	$p<0.05$
−1q−2b : −1q−2c	6.40E-01	N.S
−1q−2b : −1q−2d	1.66E-05	$p<0.05$
−1q−2c : −1q−2d	4.14E-04	$p<0.05$

Development • Supplementary information
