# Supplementary material for: Embryonic organizer specification in the mud snail Ilyanassa obsoleta depends on intercellular signaling
Source: Development. 2023 Nov 30;150(23):dev202027. doi: 10.1242/dev.202027 (PMC10730015; doi:10.1242/dev.202027)
Supplement: Supplementary file 1 [file develop-150-202027_review_history.pdf]

Development

Embryonic organizer specification in the mud snail *Ilyanassa obsoleta* depends on intercellular signaling

Jessica E. Wandelt, Ayaki Nakamoto, Morgan Q. Goulding and Lisa M. Nagy
DOI: 10.1242/dev.202027

Editor: Cassandra Extavour

Review timeline

First submission:	23 May 2023
Editorial decision:	21 July 2023
Rebuttal submitted:	26 July 2023
Editorial decision:	3 August 2023
First revision:	5 October 2023
Accepted:	23 October 2023

Original submission

First decision letter

MS ID#: DEVELOP/2023/202027

MS TITLE: Embryonic organizer specification in the mud snail Ilyanassa obsoleta depends on intercellular signaling.

AUTHORS: Jessica E. Wandelt, Ayaki Nakamoto, Morgan Q Goulding, and Lisa Nagy
ARTICLE TYPE: Research Report

Dear Dr. Nagy,

First, please accept my sincere apologies for the unacceptable length of time it has taken for me to gather reviews, make a decision, and relay it to you. This was due to a combination of late reviewer responses and to my own schedule, but it is not an acceptable time frame for authors like yourself who submit their work to Development, and for that I apologise.

I have now received all the referees' reports on the above manuscript, and have reached a decision. The referees' comments are appended below, or you can access them online: please go to BenchPress and click on the 'Manuscripts with Decisions' queue in the Author Area.

As you will see, the referees express considerable interest in your work, but have some significant criticisms and recommend a substantial revision of your manuscript before we can consider publication. I agree with Reviewer 3 that the real challenges to make this MS a clear illumination of a new mechanistic insight into a developmental process will be to (1) clarify how providing evidence for signaling between the early blastomeres being essential for cell fate specification adds anything new to the understanding that we already had that this was the case, given the work of Hyla Sweet many years ago; (2) more rigorously explain the variability of results that is currently explained only as variability between broods. I think that the work required to address these concerns, and the other important points raised by the reviewers about the quantification and analysis of some of the e.g. cell cycle phenotypes, goes beyond the scope of a typical major revision for Development, and therefore I don't think that recommending major revision is appropriate.

© 2023. Published by The Company of Biologists under the terms of the Creative Commons Attribution License (https://creativecommons.org/licenses/by/4.0/).

However, having evaluated the paper, I do recognise the potential importance of this work. I would therefore be prepared to consider as a new submission an extension of this study that contains new experiments, data and discussions and that address fully the major concerns of the referees. The work required goes beyond a standard revision of the paper. Please bear in mind that the referees (who may be different from the present reviewers) will assess the novelty of your work in the context of all previous publications, including those published between now and the time of resubmission.

If you decide to resubmit, please go to BenchPress and click on the 'Submit a new manuscript' link within the Author Area.

Please ensure that you click the 'This is a resubmission' checkbox, and enter the manuscript identification number shown above. I would also ask you to provide in the cover letter an explanation of the key ways in which the manuscript differs from the current submission, followed by a point-by-point response to the referees' concerns.

We do understand that the work entailed in a potential new submission is significant, and that you may prefer to submit elsewhere without further delay. Please do let us know if you decide not to resubmit to Development, so that we can close our file.

Many thanks for sending your work to Development.

Reviewer 1

Advance summary and potential significance to field

The concept of an organizer in animal development is an important one, and has been extensively studied in several different clades. This paper contributes to our understanding of how the D-quadrant organizer cell(s) in mollusks and other spiralians is specified. The last common ancestor of spiralians likely developed by a stereotypical cleavage program called spiral cleavage with a cell or group of cells in the D-quadrant serving as an organizer for establishing fates and the dorsal-ventral body axis. Previous work in spiralians suggested that in species that develop by equal spiral cleavage, an inductive event is required for specification of the organizer itself, while in species that develop by unequal spiral cleavage, asymmetric inheritance of determinants is necessary for specification of the organizer. This paper uses blastomere isolations in the unequal-cleaving mud snail Ilyanassa obsoleta to test this paradigm. Previous work in I. obsoleta had shown that the 3D blastomere, a cell that generates mesoderm and endoderm, requires asymmetric inheritance of determinants (via a polar lobe) in order to activate the MAPK cascade and become the organizer. In this paper, the authors demonstrate that not only are determinants from the polar lobe required, but additional signaling from neighboring animal-pole micromeres (both 1q and 2d), which will generate ectodermal derivatives, is required for specification of 3D as the organizer (assayed by activation of MAPK and by induction of ectodermal fates). This work provides a potential evolutionary bridge between inheritance of factors versus inductive signaling for establishment of the spiralian organizer since both are needed for organizer specification in I. obsoleta. It also provides some of the first evidence of potential asymmetry between 2nd quartet micromeres (i.e., that 2d may have different inductive properties relative to the other 2q micromeres); this is well known in annelids but not in mollusks. As such, this work makes a significant contribution to our understanding of how the organizer is established in a spiralian, which is important for comparisons with other animal taxa in order to better understand how organizers are molecularly established during development and how this process has evolved.

Comments for the author

My main suggestion is to add more details of how the different isolations were performed and when activated MAPK was assayed relative to the cell divisions/ablations. I think all of this can be added via editing the text, and I've outlined what I think is needed below. Knowing the timing of isolations and when MAPK was assayed is needed to make comparisons across experimental

© 2023. Published by The Company of Biologists under the terms of the Creative Commons Attribution License (https://creativecommons.org/licenses/by/4.0/).

treatments, especially since the timing of 3D division differed between treatments and because there was a large amount variation in which tissues were present after ablation between broods.

lines 87–89 “When quadrant founder cells (A, B, C, or D) were cultured after isolation at the four-cell stage and immunostained for active MAPK about eighty minutes after formation of their third micromeres, only the 3D cell showed a positive result, as expected (Fig. 1A-C).” Just to confirm, was this isolation carried out as described in the Methods (AB or CD first isolated at the 2-cell stage and then A or B or C or D isolated after the next division)? Alternatively, were these cells isolated at the 4-cell stage by lysing the other three cells? When was isolation of A, B, C or D performed relative to the second PL formation and reabsorption? Also, I don’t think this sentence is referencing the correct set of figure panels. The figure legend for 1B states that this is a Q macromere with the PL removed, and Fig. 1C shows D partial embryos. Are A, B and C partial embryos “data not shown”?

In Fig. 1D, E and F, were the micromeres successively removed at different time points in the same embryo? Or were micromeres removed at the same time (e.g., all micromeres were removed after birth of 3d for panel 1F)? Did the timing of micromere removal have an effect on activation of MAPK? For example, in the ~25% of D – 1d partial embryos that had activated MAPK, was 1d possibly removed later after its birth than in the ~75% of D – 1d partial embryos without activated MAPK?

In Fig. 1C’–F’, you demonstrate a delay in division of 3D in certain partial embryos. Related to the question above, when was activated MAPK assayed for these partial embryos? Since activated MAPK is present only in 3D in whole embryos (i.e. not in its precursor cell and not in its progeny), were the partial embryos fixed at a specific time after birth of 3D? In lines 88–89, you mention that activated MAPK was assayed 80 min after birth of the 3rd-quartet micromeres in the A, B, C and D isolates, but I couldn’t find any additional information in the Methods on the timing of fixation for the other partial embryos. When was activated MAPK assayed for Figs. 2 and 3? Were any earlier or later timepoints after birth of the 3rd-quartet micromeres assayed for activated MAPK? Is it possible that MAPK activation in the partial embryos lacking micromeres was less stable (i.e. only persisted for a short time before being degraded), was slower to accumulate, or occurred later because of the increased time before division of 3D?

For the interbrood variation depicted in Fig. 3F, is there much brood-to-brood or within brood difference in the timing of cell divisions at the same temperature? For example, when comparing two broods, was there a difference in % phenotypes in animals that had 1q micromeres removed within 5 min of their birth versus 20 min after their birth? For a single brood, were isolations performed continuously until birth of 2q micromeres (or 2d), or were they all performed within a set time window?

Additional minor comments:
> Introduction
line 52 “…founder cells, which are…”

line 61 define “Lophotrochozoa”, i.e., which phylogeny or group of taxa are you referring to with this term?

> Results and Discussion
line 157 “….ablation of 2d severely worsened…” Also, I might reword “the body-patterning performance”

For the partial embryos in which 1q was removed, 59% had activated MAPK in 3D but 100% had activated MAPK in the remaining micromeres. Furthermore, 52% of these embryos lacked a shell and foot. Taken with the results of your previous study suggesting that activation of MAPK in micromeres is necessary for their fates, does this suggest that activation of MAPK in the micromeres is not sufficient for their fates and that another factor is also needed?

lines 172–173 “Our data support a model in which 3D specification depends on signaling from multiple micromeres, whose signaling activities could be simply additive, normally summing to surpass an activation threshold” In line with this, is it possible that the surface area between

© 2023. Published by The Company of Biologists under the terms of the Creative Commons Attribution License (https://creativecommons.org/licenses/by/4.0/).

specific micromeres and 3D changes after ablation of other micromeres? How much does the positioning of the remaining micromeres with respect to 3D change after ablation?

> Figures
It would help to have more descriptive labels for each type of isolation in the figures themselves. Also, adding the “n” for the numbers of partial or whole embryos with activated MAPK to the figure panels in Figs. 1–3 (or providing this information in a table) would make it easier to compare across treatments.

For Fig. 3, which micromeres in the whole embryo have activated MAPK? Does this pattern change in embryos with 1d or 2d removed? The pattern in Fig. 3A looks different than the pattern in 3B and 3C, but I can’t tell if this is just due to rearrangement of cells after removal of a micromere. Also, is there any indication that the intensity of the signal for activated MAPK changes (e.g., the staining in Fig. 3B looks much darker than the staining in 3A).

I think it would be helpful to include a few more sentences at the end of this section discussing the broader implications of having both autonomous and conditional specification of the organizer in one species. Does this potentially indicate that both mechanisms may have been present in some last common ancestor of spiralians (or annelids + mollusks)? In lines 223–225, you state, “It seems that in different caenogastropod lineages, an ancestral mechanism conditionally specifying 3D has been supplemented or supplanted by segregated factors in vegetal cytoplasm that bias or override selection.” What is the evidence or reasoning for having conditional specification of the organizer as ancestral rather than conditional plus autonomous?

> Materials and Methods
There are some grammatical mistakes in this section (e.g., use of hyphens instead of en dashes for ranges, no space between numbers and SI unit in some places).

For removal of the PL, did agitation result in the PL falling off or was it lysed with a needle? This information is important as failure to remove part of the PL could influence the results of the study. Also, did you try removing the PL at the 4-cell stage?

Some of the ablations for Fig. 2 seem very difficult to do using a needle. How was the cellular debris from the lysed cell(s) including cortical material removed?

line 232 “Ilyanassa obsoleta obtained….”

line 241–242 I might replace the terms “killed” and “carcass” with “lysed” and “cellular remnants” in this sentence. The same goes for the following sentences.

lines 245–246 Add another sentence explaining what happened to the PL.

lines 246–247 It’s unclear how isolation described in this sentence is different than C, D or A, B isolations. Is this being done after PL removal? If so, I might add, “Following PL removal,….” to the beginning of this sentence.

line 263 “…with 80% glycerol: 1x PBS for imaging.”

line 266 “In immunohistochemical preparations the cell nuclei were visualized by DAPI staining.”

<u>Reviewer 2</u>

Advance summary and potential significance to field

The manuscript by Wandelt et al. describes embryological experiments that show that interactions with micromeres and asymmetric cytoplasmic inheritance via the polar lobe mechanism are required to specify the 3D organizer in Ilyanassa. The experimental data presented here are flawed, though not unfixable. On the other hand, oversimplification in the background introduction has undervalued this work as these experiments can potentially provide significant insights into the evolution of the D quadrant specification mechanism among spiralians. Should these issues be

© 2023. Published by The Company of Biologists under the terms of the Creative Commons Attribution License (https://creativecommons.org/licenses/by/4.0/).

addressed in the revision, this manuscript would be a valuable addition to the literature on spiralian embryology.

Comments for the author

Major Points:
1. There is a great diversity in the D quadrant specification mechanisms among spiralian phyla; this diversity is not adequately addressed in this manuscript.
In particular, the phylogenetic distribution pattern of the three major variants in the D quadrant specification (namely, equal cleavage, unequal cleavage, polar lobe) would predict some level of similarity between these three modes of D quadrant specification mechanisms, and this is consistent with the major findings presented in this manuscript. The present version has only touched on these issues briefly at the end of the discussion section and confined the discussion to molluscs. Should the diversity and its implication be appropriately addressed in the introduction, readers should be able to appreciate better the value of the experiments presented in this manuscript.

2. Please perform statistical tests on quantitative data, such as those described in lines 99-103, 113-118, and 120-126.

3. The effects of micromere ablation on the 3D cell cycle are interesting but difficult to interpret. It is necessary to demonstrate that cell cycle lengthening is specific to 3D, not a general effect on all blastomeres. Does micromere ablation affect the cell cycle lengths of 3A-3C?

4. For the bar graphs showing cell cycle lengths in different experimental conditions in Figs. 1 & 2, I believe the cell cycle lengths to be averaged numbers. However stereotyped it is, a slight variation is expected among a population. Therefore, wouldn't it be better to show the average with standard deviation, or simply plot a single embryo's 3D cell cycle length as a dot to reveal the full range of variability or the lack of variability?

5. Crepidula embryo has a tiny polar lobe, and its patterning is more similar to equal cleaver. If one lines up these results from different species, including this study, there is a clear continuity in the role of micromere-macromere interaction in second-axis patterning. Could it be that the so-called cytoplasmic inheritance does not work "chemically" (i.e., containing some transcript encoding a signaling molecule or transcription factor for D quadrant specification) but "physically" (i.e., bulking the size of one blastomere at the four-cell stage and making it more accessible for the D fate inducing signal from micromeres)? This idea is also consistent with the embryological experiment reported by Freeman & Lundelius, 1992.

Minor Points:
1. Lines 52-54: The evidence that the four quadrants are equipotent is not strong since it is difficult to assay for the condition of cell fate specification in the D quadrant before the formation of the organizer especially among the unequal cleavers. Clement's 1956 paper showed that isolated A, B, and C blastomeres developed similarly, but isolated D was clearly different. To experimentally demonstrate that the four quadrants are equipotent one would have to show that isolated A, B, and C can be induced into D fate, and that removing that induction signal would convert D into an A-, B-, or C-like fate. Polar lobe removal does make all four quadrants develop like A-C in normal embryos, but it is an interference of normal asymmetric cell division leading to the formation of blastomere D, and hence the lobe-less embryos can be interpreted as being without a D quadrant. This is inconsistent with the equipotent model because the polar lobe ablation experiment implies that the D blastomere is specified by asymmetric cytoplasmic inheritance rather than an inductive interaction with other blastomeres.

2. Lines 54-56: Why not say, "The crucial selection of just one macromere as 3D depends on signaling from first-quartet micromere derivatives (head precursor cells) surrounding the animal pole."

3. Line 122: "in all cases 3D divided with 20 minutes of controls," with or within?

4. Line 139: Please name the cells whose MAPK activation is 3D-dependent in these experiments.

© 2023. Published by The Company of Biologists under the terms of the Creative Commons Attribution License (https://creativecommons.org/licenses/by/4.0/).

5. Line 214: Taxa names such as Neogastropoda and Caenogastropoda require further explanation. Most potential readers probably don't know Neogastropoda is a subtaxon of Ceanogastropoda.

Reviewer 3

Advance summary and potential significance to field

This paper attempts to address an outstanding question about MAPK immunoreactivity in a snail embryo. Unfortunately the data presented in this paper offer little insight into this old finding or how cell fate specification occurs in spiralian embryos.

Comments for the author

This is a review of a manuscript entitled "Embryonic organizer specification in the mud snail Ilyanassa obsoleta depends on intercellular signaling" submitted to the journal Development.

This manuscript is not written in a way the average reader would appreciate the significance of the problem. There is some sloppy scholarship specifically not citing previous relevant work. In addition, readers will not likely appreciate that there are multiple forms of unequal cleavage that do not involve a polar lobe and many species of 'spiralian' exist that do not even show the canonical spiral cleavage program. The average reader will not understand that the "real" reason for writing this paper is to try and explain an observation published over 20 years ago by this lab that has been difficult to explain. Namely that the D cell is immune-reactive to an antibody to activated MAPK (actually Erk). Activation of this pathway, later found in equal cleaving invertebrates, signifies the 'response' to a signaling system and does not represent the signal itself. This result "makes sense" in equal cleaving forms in which the D cell is determined by interactions with overlying micromeres, but was difficult to interpret in unequal cleaving forms in which the D cell clearly inherits an asymmetric distribution of developmental potential.

The authors perform a limited series of identified cell deletion experiments to argue that there is 'residual' importance of overlying micromeres in D quadrant specification; however, their results are confusing and often contradictory to their own thesis. For example, in some experiments, nearly 100% of their deletions confirm previously published results (e.g. Fig. 4F, 1 and 5), which they argue are not true and represent 'batch to batch' variation. Other inconsistencies in their data are not addressed, such as the changing patterns of MAPK immunoreactivity the animal hemisphere (e.g. Fig 3B and 3D).

Furthermore, the authors make no attempt to understand the mechanism of D quadrant specification. No molecular approaches are included (what would the micromeres be secreting/doing that would influence D quadrant specification? is this the same as that expressed in unequal cleaving forms?) nor is there a morphometric analysis looking at cell contacts during early or mid-stages of development. It is known (or published anyway) that not all first quartet micromeres in Ilyanassa are equivalent, yet these possibilities are not pursued. There are published papers looking at the temporal dynamics of the induction of the D cell in other systems that the authors do not address (could it be the sum length of time of micromere interaction?). In the words of the authors, their own data "rules out a strict requirement for first-quartet cells per se" which was previously published by Sweet (1998). So what new mechanistic understanding of this process do we have from this study? Their explanation that "The between-brood variability may reflect genetic variation among wild snails, phenotypic plasticity in response to maternal or embryonic stress or simply stochastic variation in micromere signaling or 3D receptivity" comes across as hand waving, does not eliminate any possibilities of what is happening during these early embryonic events and leaves us with no new mechanistic insight in what is happening during the development of this species.

Their interpretation of the data may, or may not, support the residual role micromeres might have in the presumed ancestral initial D cell selection process selection process but the experimental evidence is extremely fragile and their own data often does not support their conclusions. There seems to be good evidence for 'developmental systems drift' in some of the molecular pathways in

© 2023. Published by The Company of Biologists under the terms of the Creative Commons Attribution License (https://creativecommons.org/licenses/by/4.0/).

this group of animals, and I am surprised that authors did not embrace this literature to help explain the variability in their results.

In summary, the data presented in this manuscript does not add any new information regarding the mechanism of cell fate determination or even the evolution of devolopment and is thus not suitable for publication in Development.

Rebuttal letter

We would like to provide the following rebuttal of our rejection and request the option of submitting a revised manuscript. Reviewer 1 has only very minor and valid points, easily addressed. Reviewer 2 calls the data "flawed, but fixable"; their noted flaws revolve almost entirely around statistical analyses which can be provided. The third review is the most critical, however, Reviewer 3 seems to have misunderstood the study's main scientific questions and has major misconceptions of both our work and the existing literature. A number of things said in their review were logically flawed, or just plain false, while at the same time often inappropriate, and objectionable in tone and style. We are unsure why the editor has chosen to give Reviewer 3 so much more weight than the other two reviews, or why an additional review was not sought due to the imbalance of opinion.

We first address the two key points Dr. Extavour notes as needing to be addressed in her rejection letter:
(1) clarify how providing evidence for signaling between the early blastomeres being essential for cell fate specification adds anything new to the understanding that we already had that this was the case, given the work of Hyla Sweet many years ago;

(2) more rigorously explain the variability of results that is currently explained only as variability between broods.

Point #1: The results of Sweet (1998) showed no evidence of intercellular signaling **whatsoever**. We repeated Sweet's 1q ablation and **we got a different result** (see Point #2). Based on Sweet's published results, the scientific community did not have a clue that 3D activation in *Ilyanassa* depends on intercellular signaling. Therefore, in contrast to every textbook that has covered this topic and many previously published manuscripts, our work shows that organizer cell fate in the classic snail model system Ilyanassa requires MORE than the inheritance of the polar lobe. Our results correct a fundamental misunderstanding in spiralian development. How the 3D cell is activated has been an open question since the 19th century. Our work transforms the existing models for 3D activation. Both Reviewers 1 and 2 understood this.

The editor's statement is in truth completely mystifying. Our best guess is that it was based on this out-of-context quote from Reviewer 3: "**In the words of the authors, their own data "rules out a strict requirement for first-quartet cells per se" which was previously published by Sweet (1998).** Reviewer 3 extracted this quote from a larger section in the manuscript explaining the complexity of the cellular origins of the signal. By isolating this phrase out of the context of the whole argument in that paragraph, the point is completely missed. Sweet's result indicated that first-quartet micromeres (1q) **are not** required for the development of 3D, and thus implied that 3D **does not receive any signal from 1q.** Our results overturn this view. The new evidence suggests that 3D organizer activation depends on signaling from overlying micromeres, but that the signal is derived from multiple cellular sources acting additively, with no one cell (or quartet) being strictly required. It remains unclear why Reviewer 3 chose to ignore the remaining part of the quoted sentence, which by doing so allowed them to draw a false parallel between our results and Sweet's.

Point #2: Explain the variability

We believe this also stems from another of Reviewer 3's misinterpretation of our results. Reviewer 3 comments: "**For example, in some experiments, nearly 100% of their deletions (in embryos**

© 2023. Published by The Company of Biologists under the terms of the Creative Commons Attribution License (https://creativecommons.org/licenses/by/4.0/).

from a single brood) confirm previously published results (e.g. Fig. 4F, 1 and 5), which they argue are not true and represent 'batch to batch' variation."

Reviewer 3 is misrepresenting us: saying that we said something which we did not say. Nowhere do we state or imply that the above-mentioned results, or any other observed phenomena, are 'not true.' Those results which reproduce the previously published ones are presented as facts, prominently, side by side with results of parallel replicates of the experiment. What Reviewer 3 calls "'batch to batch' variation" (the reviewer's words, not ours) is an interesting fact which we present quite transparently, and we clearly discuss why it's interesting. Neither Reviewer 1 or 2 raised any objection to the reported inter-brood variability.

Reviewer 3 also later comments "**Their explanation that "The between-brood variability may reflect genetic variation among wild snails, phenotypic plasticity in response to maternal or embryonic stress or simply stochastic variation in micromere signaling or 3D receptivity" comes across as hand waving, does not eliminate any possibilities of what is happening during these early embryonic events and leaves us with no new mechanistic insight in what is happening during the development of this species."**
The inter-brood variability is reported because (1) it is true; (2) it offers an explanation for why Hyla Sweet missed the inductive action of 1q; and (3) because it is biologically relevant and interesting: individual variation among wild-collected organisms (vs the isogenic lines of most model systems) is real, commonly encountered, under-reported, and certainly worthy of exploration. The variation most likely reflects physiological differences among different clutches of eggs, and quite possibly among different mother snails, and thus reveals a biological effect of potential evolutionary or ecophysiological importance.

We too would like to understand in molecular detail what happens in individual embryos that respond differently to removal of the first-quartet cells. More basically, we hope we live to see a biochemical explanation of 3D induction in any spiralian embryo. This intercellular signaling event, first deduced in the 1970s, is still almost entirely mysterious - the only chemical clues are sensitivity to monensin and brefeldin A, implicating the action of some secreted glycoprotein(s). Further functional analysis of the newly discovered signaling event in *Ilyanassa* is well beyond the scope of our study. The three non-exclusive general explanations for the observed interbrood variability we offer make quite testable predictions. The question of the source of this variability, however, is quite peripheral to the major findings of our study.

We also raise a final question about editorial fairness and appropriate reviewer tone. We feel that Reviewer3 has unnecessarily taken quite an accusatory and aggressive tone. All of us within the developmental biology and wider scientific community are working toward improving the editorial process. Logical and scientific flaws can and should be uncovered and commented on without excessive negativity. We hope that all journals are moving toward promoting that behavior in various and creative ways.

Although a request for allowing a re-submission is not usually the place to outline a rebuttal in detail, we add below a list of the most problematic statements from Reviewer 3, that further support our request.

We thank you in advance for your quick response to our request.
Thanks for your consideration.

With best wishes,
Drs Nagy, Goulding, Nakamoto and Wandelt

We have grouped the many individual points Reviewer 3 makes into 4 categories.
Reviewer 3 points are in bold, followed by our responses.

Category 1: Our hidden agenda. The reviewer repeatedly suggests, directly and indirectly, that we are secretly motivated to find the signal that activates MAPK in the 3D in *Ilyanassa*. Many of their critical comments fall under this category.

© 2023. Published by The Company of Biologists under the terms of the Creative Commons Attribution License (https://creativecommons.org/licenses/by/4.0/).

General Response: That is simply not true. We report a novel cellular mechanism for activation of the 3D cell in Ilyanassa. No secrets.

Specific responses:
"The average reader will not understand that the "real" reason for writing this paper is to try and explain an observation published over 20 years ago by this lab that has been difficult to explain. Namely that the D cell is immune-reactive to an antibody to activated MAPK (actually Erk)."
The purpose of this paper is NOT to 'explain [Erk immunoreactivity].' The purpose is to define cellular sources of an inductive signal, and to document the developmental consequences of removing those signaling sources. It is both amusing and insulting that Reviewer 3 seems to know the "real" reason for writing this paper, and tells us that we are unsuccessfully trying to hide that secret motivation.

"This paper attempts to address an outstanding question about MAPK immunoreactivity in a snail embryo."
This statement is also not true. Our study addresses the question of how specification of the organizer cell 3D is influenced by other cells. Activated MAPK is used simply as a marker for 3D specification. It is considered a reliable - i.e., biologically meaningful - marker for organizer function, precisely because pharmacological interference with MAPK phosphorylation (specifically at the time it is observed in 3D) is known to abolish 3D organizer function. Our observation of failed MAPK activation following micromere ablation correlates with loss of organizer-dependent ectodermal fates.

"Activation of this pathway, later found in equal cleaving invertebrates, signifies the 'response' to a signaling system and does not represent the signal itself."
Within spiralian systems, nobody knows what active Erk does; nor does anyone understand how it is activated. The statements made so confidently about what this pathway 'signifies' and 'represents' are unfounded. Incidentally, the reviewer's choice of words ('equal cleaving invertebrates') may be a typo or may betray a lack of familiarity with spiralians and other invertebrates. "Equal cleaving spiralians" is a meaningful term; "equal cleaving invertebrates" is not.

"This result "makes sense" in equal cleaving forms in which the D cell is determined by interactions with overlying micromeres, but was difficult to interpret in unequal cleaving forms in which the D cell clearly inherits an asymmetric distribution of developmental potential."
The functional significance of MAPK activation in 3D in any spiralian embryo is not yet well understood, so it makes as much and as little sense as any other enzyme regulation event whose function is poorly understood, both in equal cleaving and unequal cleaving forms. Erk activation in the 3D cell of *Ilyanassa* is not 'difficult to explain' in the sense that it does not contradict or challenge any theoretical framework within spiralian embryology. Without directly saying it, the reviewer assumes MAPK must be activated in response to an extracellular signal, as it commonly is. It is known that MAPK can be activated by intracellular changes in [Ca2+] or by other means of autocrine signaling. We want to emphasize that Reviewer 3 is building an argument that is logically unfounded and simply not relevant to our study. Our results now better position future experiments aimed at finding whatever it is that activates MAPK, external or internal to 3D.

Category 2: Insufficient, contradictory, inconsistent data.
General Response: A very extensive set of (technically challenging) experiments were done: this is the first study to systematically test simultaneous ablation of micromeres in multiple tiers (first three quartets) in any mollusk. None of our data is contradictory or inconsistent. Again, these criticisms are unique to Reviewer 3.

Specific responses:
"The authors perform a limited series of identified cell deletion experiments to argue that there is 'residual' importance of overlying micromeres in D quadrant specification…"
As noted above, it is important to realize we are not reporting a trivial amount of work. In addition, there is nothing residual about the signal we have identified. 'Residual' is the reviewer's term. Variable yes, but not residual. This again is the reviewer's misconception of our reported variability.

© 2023. Published by The Company of Biologists under the terms of the Creative Commons Attribution License (https://creativecommons.org/licenses/by/4.0/).

“ inconsistencies in their data are not addressed, such as the changing patterns of MAPK immunoreactivity the animal hemisphere (e.g. Fig 3B and 3D).”
It’s true that we do not provide a detailed analysis of cell-specific MAPK immunoreactivity dynamics among the roughly twenty cells of what Reviewer 3 oddly calls the ‘animal hemisphere’ (micromeres and micromere daughter cells). A dynamic pattern was described qualitatively, with unfortunately low temporal resolution, by Lambert & Nagy (2001) in the paper that provided the first report of organizer-linked MAPK activation in any spiralian embryo. Because that low-resolution study has not yet been followed by a more exhaustive descriptive analysis, a well-defined pattern is not very predictable; nobody knows what the real pattern is. Characterizing this pattern and its variability is not the aim of the present study, and we do not imagine that such an analysis would contribute one way or another to the important conclusions drawn.

Category 3: Sloppy scholarship, implications that the authors don’t know the relevant literature, and why haven’t the authors completed, or even mentioned x, y and z.

General response: Not all relevant literature can be addressed in a word-limited submission, nor is there space to describe all relevant next experiments that our results open the door to. That does not equate with sloppy scholarship.

Specific responses:
“There is some sloppy scholarship specifically not citing previous relevant work.”
The reviewer fails to mention what they are referring to. The manuscript is extensively and appropriately referenced. An accusation of sloppiness from a reviewer should be better supported.

“It is known (or published anyway) that not all first quartet micromeres in Ilyanassa are equivalent, yet these possibilities are not pursued.”
First, some of these possibilities were pursued. Second, this fact is well known to us; indeed, one of us (MQG) published a 2003 article in Development with an extensive experimental analysis of the causes and consequences of 1d non-equivalence within the first quartet in *Ilyanassa*. For the present study, we focused mainly on first-quartet cells collectively, essentially repeating the major experiment that revealed 3D induction in equal-cleaving gastropods and a polyplacophoran. Additionally, some of our experiments test the effect of ablating the 1d cell alone or in combination with 2d.

“There are published papers looking at the temporal dynamics of the induction of the D cell in other systems that the authors do not address (could it be the sum length of time of micromere interaction?).”
We are aware of the study published in 2017 by Henry, Lyons, Perry and Osborne, in which the whole first quartet (or one or the other tier of daughter cells) was laser-ablated at various time windows; this study followed up on the finding (reported in 2006) that 1q ablation abolishes 3D specification in *Crepidula* . Now that a 3D-inducing signal has been discovered in *Ilyanassa*, analysis of temporal dynamics will be just one of many interesting sets of further experiments to do. It does not seem to us necessary to include such an analysis in the present study.

“...nor is there a morphometric analysis looking at cell contacts during early or mid-stages of development.”
As Reviewer 3 may not know, such a morphometric analysis has already been completed, in a qualitative way, by Hyla Sweet (unpublished Ph.D. thesis); we have seen the results, so we know which micromeres are reproducibly in contact with the D macromere from the 8-cell to the 24-cell stage in I*lyanassa*.

Category 4: No new mechanism, no new molecules

General response: Again, Reviewer 3 seems stuck on the idea that the only interesting data we could possibly present on *Ilyanassa* development is the actual molecule that induces MAPK activation in 3D. We argue that the identification of a cellular signaling mechanism, which we have done, does not necessitate identification of the underlying molecular signal in the same research report.

Specific responses:

© 2023. Published by The Company of Biologists under the terms of the Creative Commons Attribution License (https://creativecommons.org/licenses/by/4.0/).

"Furthermore, the authors make no attempt to understand the mechanism of D quadrant specification. No molecular approaches are included (what would the micromeres be secreting/doing that would influence D quadrant specification? is this the same as that expressed in unequal cleaving forms?)"
We did not look for the underlying MOLECULAR mechanism, and argue that many papers published in Development do not explore the molecular basis of the cellular mechanisms they report. This is not merely descriptive work. Cell ablations are a well-respected functional assay. Our work provides valuable insight for the next group of scientists to work on this topic with a molecular approach. Directing attention away from the polar lobe for uncovering the molecular mechanisms is an invaluable contribution to the field.
We will be delighted when future investigators succeed in characterizing the molecular mechanisms of spiralian organizer specification and function, and even more delighted when further study finds out if the same or different kinds of stuff is secreted in various equal-cleaving forms (including gastropods, polyplacophorans, annelids, flatworms et cetera) and in unequal cleavers of distantly related clades. Or better yet, that it's something other than the expected secreted signaling molecule. It's going to be a lot of work, and a lot of fun, and the results presented here will help direct that research.

"There seems to be good evidence for 'developmental systems drift' in some of the molecular pathways in this group of animals, and I am surprised that authors did not embrace this literature to help explain the variability in their results."
We are well aware of the very surprising disparity found in organizer-linked molecular signaling factors between *Ilyanassa* and *Crepidula*; given the nearly identical patterns of early cell division and later embryogenesis in these taxa, it seems likely that major DSD has occurred over the one or two hundred million years since these lineages diverged. We look forward to finding out more about what changes have happened. Meanwhile, developmental variation within a single population in one species is another thing - also interesting, but not clearly related, and not explicable by the scant knowledge we have of long-term DSD in any spiralian taxa.

"In summary, the data presented in this manuscript does not add any new information regarding the mechanism of cell fate determination or even the evolution of development and is thus not suitable for publication in Development."
We submit that by revealing the existence of an intercellular signaling event which had previously been ruled out, we are adding novel and essential information regarding mechanisms of cell fate determination.

Rebuttal decision letter

MS ID#: DEVELOP/2023/202027

MS TITLE: Embryonic organizer specification in the mud snail Ilyanassa obsoleta depends on intercellular signaling.

AUTHORS: Jessica E. Wandelt, Ayaki Nakamoto, Morgan Q. Goulding, and Lisa M. Nagy

Dear Dr. Nagy,

This is the formal invitation to submit a revised manuscript. For details, please see my previous correspondence to you about this - if you have not received this under separate cover, then please let me know.

If you choose to submit a revision, please feel free to respond/implement/discuss suggestions and comments only of Reviewers 1 and 2. There is no need to formally respond to the comments of Reviewer 3.

To submit a revision, please go to your Author Area and click on the 'Submit a Revision' link.

© 2023. Published by The Company of Biologists under the terms of the Creative Commons Attribution License (https://creativecommons.org/licenses/by/4.0/).

First revision

Author response to reviewers' comments

These replies to the reviewers will also be uploaded as a PDF with the manuscript files.

The authors thank the reviewers for the helpful suggestions. The manuscript is improved through your efforts!

Reviewer 1 Comments for the Authors
Reviewer 1: My main suggestion is to add more details of how the different isolations were performed and when activated MAPK was assayed relative to the cell divisions/ablations. I think all of this can be added via editing the text, and I've outlined what I think is needed below. Knowing the timing of isolations and when MAPK was assayed is needed to make comparisons across experimental treatments, especially since the timing of 3D division differed between treatments and because there was a large amount variation in which tissues were present after ablation between broods.
Response: All micromere ablations were done within the same narrow time window: during the first 20-25% of the cell cycle. After this time interval, compaction makes micromeres very hard to lyse by freehand needle puncture. In all experiments, operations were begun as early as possible and continued as long as possible, for the full duration of that early-interphase time interval. Most of this was in the original methods, but we've added a few more details. It therefore seems unlikely that variation in ablation timing can explain the different results obtained in different broods.
As for the timing of fixation for phospho-MAPK staining, see reviewer comment and response below.

Reviewer2: lines 87-89 "When quadrant founder cells (A, B, C, or D) were cultured after isolation at the four-cell stage and immunostained for active MAPK about eighty minutes after formation of their third micromeres, only the 3D cell showed a positive result, as expected (Fig. 1A-C)." Just to confirm, was this isolation carried out as described in the Methods (AB or CD first isolated at the 2-cell stage and then A or B or C or D isolated after the next division)? Alternatively, were these cells isolated at the 4-cell stage by lysing the other three cells? When was isolation of A, B, C or D performed relative to the second PL formation and reabsorption?
Response: Yes, these cells were isolated as described in the methods. Separated at the 2-cell stage, then separated after the next division. They were not isolated by lysing the other three cells.

Reviewer 3. Also, I don't think this sentence is referencing the correct set of figure panels. The figure legend for 1B states that this is a Q macromere with the PL removed, and Fig. 1C shows D partial embryos. Are A, B and C partial embryos "data not shown"?
Response: We believe the cause of confusion here is in the original figure legend. That figure legend said "(B) Absence of activated MAPK in unidentified quadrant ('Q') isolated after polar lobe removal." We have replaced this with '...unidentified non-D quadrant isolate (A, B, or C).'

Reviewer 4: In Fig. 1D, E and F, were the micromeres successively removed at different time points in the same embryo? Or were micromeres removed at the same time (e.g., all micromeres were removed after birth of 3d for panel 1F)? Did the timing of micromere removal have an effect on activation of MAPK? For example, in the ~25% of D - 1d partial embryos that had activated MAPK, was 1d possibly removed later after its birth than in the ~75% of D - 1d partial embryos without activated MAPK?
Response: We have added the sentence "Micromeres were removed in rapid succession during this time window." to the 'Microsurgery' section in Materials and Methods, to further clarify the procedure. We did not test whether the removal at 5 min, for example, differed from removal at 25 min.

© 2023. Published by The Company of Biologists under the terms of the Creative Commons Attribution License (https://creativecommons.org/licenses/by/4.0/).

Reviewer 5: In Fig. 1C'–F', you demonstrate a delay in division of 3D in certain partial embryos. Related to the question above, when was activated MAPK assayed for these partial embryos? Since activated MAPK is present only in 3D in whole embryos (i.e., not in its precursor cell and not in its progeny), were the partial embryos fixed at a specific time after birth of 3D? In lines 88-89, you mention that activated MAPK was assayed 80 min after birth of the 3rd-quartet micromeres in the A, B, C and D isolates, but I couldn't find any additional information in the Methods on the timing of fixation for the other partial embryos. When was activated MAPK assayed for Figs. 2 and 3? Were any earlier or later time points after birth of the 3rd-quartet micromeres assayed for activated MAPK? Is it possible that MAPK activation in the partial embryos lacking micromeres was less stable (i.e., only persisted for a short time before being degraded), was slower to accumulate, or occurred later because of the increased time before division of 3D?
Response: Fixation was done at a standard time in all cases: 80' after the median time of 3q formation. The timing of 3q formation is quite invariant among all the situations here. These details are in the Materials and Methods. It is indeed possible that after micromere ablation, 3D activates MAPK at an abnormally late stage, or with an abnormally brief duration. The important point made here is that normally, phospho-MAPK appears at a very predictable time and reliably persists for a good long while, and that following micromere ablation, this reliable and persistent activation isn't observed.

Reviewer 6: For the interbrood variation depicted in Fig. 3F, is there much brood-to-brood or within brood difference in the timing of cell divisions at the same temperature? For example, when comparing two broods, was there a difference in % phenotypes in animals that had 1q micromeres removed within 5 min of their birth versus 20 min after their birth? For a single brood, were isolations performed continuously until birth of 2q micromeres (or 2d), or were they all performed within a set time window?
Response: In our hands, 1q ablation was performed until the onset of the "compaction stage," after which micromeres flatten out and become resistant to needle puncture. Compaction occurs in a cell-cycle dependent manner, thus our 1q ablation experiments were performed within a similar time window of the cell cycle. Although it is likely that different results in Fig. 4F resulted from the difference of capsules (brood-to-brood), rather than the timing of ablation (within brood difference), we cannot eliminate the reviewer's suggestion that a 15-minute difference in ablation time could make a difference. To acquire any sort of within brood replicates that type of ablation efficiency might require a very steady-handed robot! Nonetheless, we recognize the reviewer's suggestion and have added the sentence: "However, it is also possible that minor variations in the timing of the micromere ablations contributed to the variation observed. "

Reviewer: Additional minor comments:
> Introduction
Reviewer 7. line 52 "…founder cells, which are…"
Response: Changed: 'In these embryos, symmetric cell divisions in the first two rounds of mitosis produce four body-quadrant founder cells that show no difference in potential upon experimental challenge.'

Reviewer 8: line 61 define "Lophotrochozoa", i.e., which phylogeny or group of taxa are you referring to with this term?
Response: In the revised document we stick with the term Spiralia to refer to the superphylum that includes the mollusks.

Reviewer:> Results and Discussion
Reviewer 9. line 157 "…. ablation of 2d severely worsened…" Also, I might reword "the body-patterning performance"
Response: changed as suggested

Reviewer 10: For the partial embryos in which 1q was removed, 59% had activated MAPK in 3D but 100% had activated MAPK in the remaining micromeres. Furthermore, 52% of these embryos lacked a shell and foot. Taken with the results of your previous study suggesting that activation of MAPK in micromeres is necessary for their fates, does this suggest that activation of MAPK in the micromeres is not sufficient for their fates and that another factor is also needed?

© 2023. Published by The Company of Biologists under the terms of the Creative Commons Attribution License (https://creativecommons.org/licenses/by/4.0/).

Response: Yes, absolutely to both points.
Our key finding is that MAPK in 3D requires an early micromere signal. The data also implicates two other signaling events: 1) MAPK activation in the later micromeres depends on the polar lobe, but this is a different, or redundantly acting, signal than one controlled by 3DMAPK and 2) MAPK activation in the micromeres is necessary but not sufficient for their fates, thereby suggesting other signaling events are at play. We have added a few words about these implications for additional signaling.

Some canonical signaling pathways have been implicated in regulation of micromere fate in *Ilyanassa*: dpp/BMP4 regulates eye, shell, foot neurogenic ectoderm fate (Lambert et al. 2016); Notch is involved in 4d inductive signaling (Gharbiah et al. 2014), and many more will likely be uncovered .

Reviewer 11: lines 172-173 "Our data support a model in which 3D specification depends on signaling from multiple micromeres, whose signaling activities could be simply additive, normally summing to surpass an activation threshold" In line with this, is it possible that the surface area between specific micromeres and 3D changes after ablation of other micromeres? How much does the positioning of the remaining micromeres with respect to 3D change after ablation?

Response: This is an interesting question and deserves to be addressed with high-resolution imaging of cell surface interfaces in intact and operated embryos, ideally combined with visualization of MAPK activation state in live embryos! Such an analysis is well beyond the scope of the present study, whose main purpose is just to test the requirement for extracellular signaling in 3D specification, and to identify the sources of that signaling.

Reviewer 12:> Figures
It would help to have more descriptive labels for each type of isolation in the figures themselves. Also, adding the "n" for the numbers of partial or whole embryos with activated MAPK to the figure panels in Figs. 1-3 (or providing this information in a table) would make it easier to compare across treatments.

Response: We have added the numbers of partial or whole embryos with activated MAPK to the figure panel, and additional labels about which cells were ablated.

Reviewer 13: For Fig. 3, which micromeres in the whole embryo have activated MAPK? Does this pattern change in embryos with 1d or 2d removed? The pattern in Fig. 3A looks different than the pattern in 3B and 3C, but I can't tell if this is just due to rearrangement of cells after removal of a micromere. Also, is there any indication that the intensity of the signal for activated MAPK changes (e.g., the staining in Fig. 3B looks much darker than the staining in 3A).

Response: The embryos in Fig. 3 were fixed at 3q +210. As expected from patterns described in Lambert and Nagy, we can identify MAPK activity in remaining derivatives of second and third quartet cells in the A, C and D quadrants. No activation was observed in derivatives of 2b, or in the third quartet cells, 3a and 3b. As you note the precise arrangements of cells changes after removal of micromeres, and we did not carry out sufficient time course analysis to precisely mark these changes, nor did we compare micromere activation, or variation in the intensity of staining, in sufficient numbers of embryos to make any firm conclusions about variability in micromere activation. The primary conclusion from this figure is the marked difference between the micromere MAPK activation in the 1q +2d ablated embryos compared to any of the other ablations.

Reviewer 14: I think it would be helpful to include a few more sentences at the end of this section discussing the broader implications of having both autonomous and conditional specification of the organizer in one species. Does this potentially indicate that both mechanisms may have been present in some last common ancestor of spiralians (or annelids + mollusks)? In lines 223-225, you state, "It seems that in different caenogastropod lineages, an ancestral mechanism conditionally specifying 3D has been supplemented or supplanted by segregated factors in vegetal cytoplasm that bias or override selection." What is the evidence or reasoning for having conditional specification of the organizer as ancestral rather than conditional plus autonomous?

Response: The expanded Introduction should satisfy the need for explaining the theory of 3D specification evolution - basically the phylogenetic mapping of equal vs unequal cleavers by Freeman & Lundelius, which is not broken by more recent resolution of phylogeny.

© 2023. Published by The Company of Biologists under the terms of the Creative Commons Attribution License (https://creativecommons.org/licenses/by/4.0/).

Reviewer 15:> Materials and Methods
There are some grammatical mistakes in this section (e.g., use of hyphens instead of en dashes for ranges, no space between numbers and SI unit in some places).
Response: changed as suggested

Reviewer 16: For removal of the PL, did agitation result in the PL falling off or was it lysed with a needle? This information is important as failure to remove part of the PL could influence the results of the study. Also, did you try removing the PL at the 4-cell stage?
Response: The PL was removed with agitation as described. We did not try removing it at second cleavage - in fact this has never been done.

Reviewer 17: Some of the ablations for Fig. 2 seem very difficult to do using a needle. How was the cellular debris from the lysed cell(s) including cortical material removed?
Response: Indeed, they were challenging ablations! We have added the sentence: "In all cases, ablated cells detach from the embryo and are easily removed by gentle pipetting within a few minutes of deletion." to further clarify the ablation process.

Reviewer 18: line 232 "Ilyanassa obsoleta obtained…."
Response: changed

Reviewer 19: line 241-242 I might replace the terms "killed" and "carcass" with "lysed" and "cellular remnants" in this sentence. The same goes for the following sentences.
Response: changed as suggested

Reviewer20: lines 245-246 Add another sentence explaining what happened to the PL.
Response: We have added the phrase: following agitation the PL comes loose immediately upon full constriction of the furrow (Collier, 1981, Lambert and Nagy 2001). Hope that is helpful? This is a standard procedure for isolating polar lobes.

Reviewer21: lines 246-247 It's unclear how isolation described in this sentence is different than C, D or A, B isolations. Is this being done after PL removal? If so, I might add, "Following PL removal," to the beginning of this sentence.
Response: changed as suggested

Reviewer 22: line 263 "…with 80% glycerol: 1x PBS for imaging."
Response: changed as suggested

Reviewer 23: line **266 "In immunohistochemical preparations the cell nuclei were visualized by DAPI staining."**
Response: changed as suggested

Reviewer 2 Comments for the Author…
The manuscript by Wandelt et al. describes embryological experiments that show that interactions with micromeres and asymmetric cytoplasmic inheritance via the polar lobe mechanisms are required to specify the 3D organizer in *Ilyanassa*. The experimental data presented here are flawed, though not unfixable. On the other hand, oversimplification in the background introduction has undervalued this work, as these experiments can potentially provide significant insights into the evolution of the D quadrant specification mechanism among spiralians. Should these issues be addressed in the revision; this manuscript would be a valuable addition to the literature on spiralian embryology.

Major Points:

1. Reviewer: There is a great diversity in the D quadrant specification mechanisms among spiralian phyla; this diversity is not adequately addressed in this manuscript. In particular, the phylogenetic distribution pattern of the three major variants in the D quadrant specification (namely, equal cleavage, unequal cleavage, polar lobe) would predict some level of similarity between these three modes of D quadrant specification mechanisms, and this is consistent with the major findings presented in this manuscript. The present version has only touched on these issues briefly at the end of the discussion section and confined the discussion of molluscs.

© 2023. Published by The Company of Biologists under the terms of the Creative Commons Attribution License (https://creativecommons.org/licenses/by/4.0/).

Should the diversity and its implication be appropriately addressed in the introduction, readers should be able to appreciate better the value of the experiments presented in this manuscript.
Response: The greatly expanded Introduction should satisfy the need for explaining the theory of 3D specification evolution - basically the phylogenetic mapping of equal vs unequal cleavers by Freeman & Lundelius, which is not broken by more recent resolution of phylogeny.

Reviewer 2: Please perform statistical tests on quantitative data, such as those described in lines 99-103, 113-118, and 120-126.
Response: On the advice of UA Stats consultants, the two-tailed Mann-Whitney *U*-test was used to compare the cell cycle lengths. The statistical tests show that cell cycle lengths were significantly delayed in the experimental groups (lines 113-8 [Fig. 1C'-1F7'], lines 120-6 [Fig.2E-2F]). The chi-squared test was used to test the significance of the MAPK activation in the D quadrant (lines 99-103). The frequency of MAPK activation was significantly decreased in Fig. 1B and Fig. 1D-F. These results are now indicated in the figures/ figure legends.

Reviewer 3. The effects of micromere ablation on the 3D cell cycle are interesting but difficult to interpret. It is necessary to demonstrate that cell cycle lengthening is specific to 3D, not a general effect on all blastomeres. Does micromere ablation affect the cell cycle lengths of 3A-3C?
Response: Good question! Unfortunately, we did not measure the effect on the other macromeres.

Reviewer 4. For the bar graphs showing cell cycle lengths in different experimental conditions in Figs. 1 & 2, I believe the cell cycle lengths to be averaged numbers. However, stereotyped it is, a slight variation is expected among a population. Therefore, wouldn't it be better to show the average with standard deviation, or simply plot a single embryo's 3D cell cycle length as a dot to reveal the full range of variability or the lack of variability?
Response:We did not collect data on each individual embryo. Micromere ablated embryos were not cultured individually, and we avoided handling them as much as possible subsequent to the ablations. The mean population time of 3D division (i.e., the fourth micromere formation) was determined by the following method: pools of micromere ablated, and control embryos were kept in separate small petri dishes and monitored every 15-20 minutes. The time when the first cells had divided in each dish was recorded, followed by the time when roughly half of the population had divided. Statistical significance of the changes in cell cycle length were calculated using the Mann-Whitney *U*-test (https://www.socscistatistics.com/ tests/mannwhitney/default2.aspx).

Reviewer 5. Crepidula embryo has a tiny polar lobe, and its patterning is more similar to equal cleaver. If one lines up these results from different species, including this study, there is a clear continuity in the role of micromere-macromere interaction in second-axis patterning. Could it be that the so-called cytoplasmic inheritance does not work "chemically" (i.e., containing some transcript encoding a signaling molecule or transcription factor for D quadrant specification) but "physically" (i.e., bulking the size of one blastomere at the four-cell stage and making it more accessible for the D fate inducing signal from micromeres)? This idea is also consistent with the embryological experiment reported by Freeman & Lundelius, 1992.
Response:This is a nice idea, but is contradicted by experiments on Bithynia (which has a pretty puny polar lobe, but apparently autonomous D quadrant specification). Anyway, the question of how polar lobes work is outside the scope of this study. It should be noted, however, that this study includes the first demonstration that PL inheritance is indeed required for 3D MAPK activation!

Minor Points:
Reviewer 1. Lines 52-54: The evidence that the four quadrants are equipotent is not strong since it is difficult to assay for the condition of cell fate specification in the D quadrant before the formation of the organizer, especially among the unequal cleavers. Clement's 1956 paper showed that isolated A, B, and C blastomeres developed similarly, but isolated D was clearly different. To experimentally demonstrate that the four quadrants are equipotent, one would have to show that isolated A, B, and C can be induced into D fate, and that removing that induction signal would convert D into an A-, B-, or C-like fate. Polar lobe removal does make all four quadrants develop like A-C in normal embryos, but it is an interference of normal asymmetric cell division leading to the formation of blastomere D, and hence the lobe-less embryos can be interpreted as being without a D quadrant. This is inconsistent with the equipotent model because the polar lobe ablation experiment implies that the D blastomere is

© 2023. Published by The Company of Biologists under the terms of the Creative Commons Attribution License (https://creativecommons.org/licenses/by/4.0/).

specified by asymmetric cytoplasmic inheritance rather than an inductive interaction with other blastomeres.
Response: It seems that Reviewer 2 is thinking we were talking about *Ilyanassa*. We weren't. Quadrant equipotency has clearly been demonstrated by experiments on embryos of *Crepidula*, *Lymnaea*, *Patella*, as well as equal-cleaving forms outside *Gastropoda*, as referenced in the Introduction. These equal cleavers are the ones meant by 'most of these snails' referenced in lines 52-4 of the originally submitted MS.

Reviewer 2. Lines 54-56: Why not say, "The crucial selection of just one macromere as 3D depends on signaling from first-quartet micromere derivatives (head precursor cells) surrounding the animal pole."
Response: changed as suggested

Reviewer 3. Line 122: "in all cases 3D divided with 20 minutes of controls," with or Within?
Response: yes, 'within', thanks for catching that

Reviewer 4. Line 139: Please name the cells whose MAPK activation is 3D-dependent in these experiments.
Response: The embryos in Fig. 3 were fixed at 3q +210. As expected from patterns described in Lambert and Nagy, we can identify MAPK activity in remaining derivatives of second and third quartet cells in the A, C and D quadrants. No activation was observed in derivatives of 2b, or in the third quartet cells 3a and 3b. As you note, the precise arrangements of cells change after removal of micromeres, and we did not carry out sufficient time course analysis to precisely mark these changes, nor did we compare micromere activation, or variation in the intensity of staining, in sufficient numbers of embryos to make any firm conclusions about variability in micromere activation. The primary conclusion from this figure is the marked difference between the micromere MAPK activation in the 1q +2d ablated embryos compared to any of the other ablations. We have included reference to the above in the legend to Figure 3.

Reviewer 5. Line 214: Taxa names such as Neogastropoda and Caenogastropoda require further explanation. Most potential readers probably don't know Neogastropoda is a subtaxon of Ceanogastropoda.
Response: Reference to Neogastropoda has been removed in the revised manuscript.

Second decision letter

MS ID#: DEVELOP/2023/202027

MS TITLE: Embryonic organizer specification in the mud snail Ilyanassa obsoleta depends on intercellular signaling.

AUTHORS: Jessica E. Wandelt, Ayaki Nakamoto, Morgan Q. Goulding, and Lisa M. Nagy
ARTICLE TYPE: Research Report

I am happy to tell you that your manuscript has been accepted for publication in Development, pending our standard ethics checks. Although this is not a condition of acceptance, I would ask that you consider adding a sentence or two in proof to address the comment of Reviewer 2 on clarifying the relationship between cell cycle length and cell fate, which I believe is a useful suggestion.

Reviewer 1

Advance summary and potential significance to field

This work is significant and reveals new variation in how a spiralian organizer can be specified, which has interesting evolutionary implications. The manuscript provides evidence of dual

© 2023. Published by The Company of Biologists under the terms of the Creative Commons Attribution License (https://creativecommons.org/licenses/by/4.0/).

autonomous and conditional mechanisms underlying organizer specification in a mollusc that develops by unequal spiral cleavage. This breaks the long-standing argument that the mechanism of spiralian organizer specification (autonomous versus conditional) is solely based on the mode of spiral cleavage (unequal versus equal). These data will be foundational to efforts in understanding how spiralian organizers are molecularly established and how this process evolved.

Comments for the author

My suggestions and questions from the first review have been addressed or answered. The added text throughout has clarified details of the isolations and timing of the assays for activated MAPK, and the additional text in the Intro and Discussion have strengthened the evolutionary implications of this work. The addition of statistical analyses for comparing cell cycle lengths and MAPK activation across treatments is also helpful. I do not have any major suggestions for further revision.
Minor comments

Intro line 47 Awkwardly worded sentence

line 77 I suggest changing “basal” to “early-branching”.

line 80 I suggest changing “cell-autonomously” to “in a cell-autonomous manner”.

Results & Discussion

The description of the experiments depicted in Fig. 1B is still confusing to me. In the results section, Fig. 1B is referred to twice. The sentence from lines 117–119, “When quadrant founder cells (A, B, C, or D) were cultured….(Fig. 1A – C)” sounds like the embryo in Fig. 1B is from an isolation of AB or CD at the two-cell stage and then removal of another cell after the next division. This is also what the figure legend (line 492) suggests to me (this is an unidentified, isolated A, B or C macromere). I interpret both of these sentences to suggest that the PL was not removed during the isolation. However, the title of this Results section (line 109) and the last sentence in this section (lines 119–120), “We also observed absence of MAPK activation in any cell of unidentified quadrants after PL removal….(Fig. 1B)”, suggests that the embryo shown in Fig. 1B is from an experiment in which the PL was removed at the trefoil stage and then one cell at the 2-cell stage was removed followed by removal of a second cell after the next division (leaving either A, B, C or D-PL, which cannot be distinguished apart because of lack of the PL). If this is the case, then I don’t think lines 117–119 should refer to Fig. 1B and the figure legend for Fig. 1B should be modified to say something like, “Absence of activated MAPK in any unidentified isolated macromere after removal of the PL at the trefoil stage.”

For the section that starts on line 183, I might add a sentence or two explaining which micromeres contribute to the foot and shell in Ilyanassa.

Methods
line 348 Font appears different.

Figures – I think the order is switched around in the pdf.

Fig. legends
lines 505 and 506 Switch “Left:” and “Right:” around.
lines 507 – 510 I would add an “s” to “embryo” throughout.

Reviewer 2

Advance summary and potential significance to field

This manuscript by Wandlet et al. describes a set of embrological experiments which demonstrate a requirement of micromere-derived inductive signal for the specification of D quadrant organizer in Illyanassa - a species that has long been considered to use cell autonomous mechanism for D quadrant specification. This finding bridges significant gap in our current understand of the

© 2023. Published by The Company of Biologists under the terms of the Creative Commons Attribution License (https://creativecommons.org/licenses/by/4.0/).

diversity and evolution of D quadrant specification mechanisms among spiralians. This revision is a great improvement over the previous version. The expanded introduction has served the purpose of bringing the described experiments into perspective. These additions would allow more readers to appreciate their important implications on spiralian embryology and the evolution of developmental mechanisms.

Comments for the author

I still have a problem using cell cycle length to indicate cell fate status in this experimental setting. As the authors have clearly documented, the lengthening of the cell cycle following blastomere isolation and micromere ablation correlates to the number of micromeres deleted. It is not an either-(D)-or-(ABC) condition but a continuum. 3D MAPK activity was detected in D quadrant isolates, but the cell cycle length was also significantly longer than normal. This means that the lengthening of the cell cycle is not a consequence of a failure in MAPK activation. As the author pointed out, it could mean that there are parallel inductive signals to determine different aspects of 3D phenotypes. However, it is also possible that cell cycle delay is a general effect of blastomere deletion. Therefore, it is imperative to demonstrate that this delay is specific to 3D. Ideally, it would be best if the same experiments could be performed on the A, B, or C quadrant. I understand that these experiments are so difficult that asking for more experiments would not be a real option. (And thus, getting this manuscript published is important not only for the authors but also for the spiralian community!) Is there any documentation of the remaining micromeres' cell cycle lengths? Such data is crucial for using cell cycle length to infer 3D identity. In many places, the authors were careful enough not to overstate the connection between cell cycle length and cell identity. However, in places such as line 100, the authors wrote “Using four independent assays of 3D identity or organizer function, we assessed…” Here, cell cycle length is clearly implicated as an assay for 3D identity (or its consequence). If the data to demonstrate the specificity of cell cycle delay are not available, a possible way to get around this issue is to be more careful with the wording throughout the text and acknowledge the possibility that cell cycle lengthening might be a general effect of cell ablation rather than an indication of non-3D fate.

Minor points:
In abstract, line 23, “some intercellular signal(s)” might be replaced with “inductive signal(s);” in line 24, replace “activating the special properties of 3D” with “normal expression of 3D-specific phenotypes.”
Line 26: It is not clear what is equalized to what.
Lines 27-28: the effects of first quartet-micromere ablation “on the expression of 3D phenotypes”
Line 95: delete “quite distantly related;” similarly in line 96, delete “distantly related” and maybe say “in yet another caenogastropod.” Phylogenetic distances are relative, and no closely related species are inferred in this paragraph, so there should be no distantly related species, either.
It would be beneficial to add a one-sentence summary/conclusion to the end of each result section, e.g., to the end of lines 120, 132, 154, 163.
It seems that most of the statistical information is given in the figure and legends now, but the sample sizes (n) are still given in the main text. For both improving readability and helping readers navigate the data, it would be nice to have all these in one place. I suggest moving N and percentage to figure (and figure legeds).

© 2023. Published by The Company of Biologists under the terms of the Creative Commons Attribution License (https://creativecommons.org/licenses/by/4.0/).
